# Redefinition of EEG frequency bands: a fractal model inspired by Blagg’s Titius–Bode law

**DOI:** 10.3389/fnsys.2026.1736474

**Published:** 2026-03-19

**Authors:** Sultan Tarlacı, Metin Çınaroğlu, Eda Yılmazer, Selami Varol Ülker

**Affiliations:** 1 Medical School, Üsküdar University, İstanbul, Türkiye; 2 Department of Psychology, Faculty of Administrative and Social Science, İstanbul Nişantaşı University, İstanbul, Türkiye; 3 Department of Psychology, Faculty of Social Science, Beykoz University, İstanbul, Türkiye; 4 Department of Psychology, Faculty of Humanities and Social Science, Üsküdar University, İstanbul, Türkiye

**Keywords:** EEG spectrum, exponential growth, Mary Adela Blagg, neurophysics, Schumann resonance, Titius–Bode Blagg law

## Abstract

The canonical frequency bands used to categorize human electroencephalographic (EEG) activity—delta, theta, alpha, beta, and gamma—have historically been defined using pragmatic and variably applied thresholds rather than a unifying mathematical principle. In this theoretical study, we propose a geometric framework for redefining EEG frequency bands based on logarithmic scaling, drawing on the exponential formulation introduced in Mary Blagg’s refinement of the Titius–Bode law. Using the mean adult alpha rhythm as a reference frequency and applying a constant scaling ratio (*R* = 1.7275), we derive a mathematically ordered hierarchy of EEG band centers and boundaries within a continuous log-spaced spectrum. Unlike descriptive models of spectral 1/f scaling, the present framework provides an explicit generative rule for discrete band centers and transition frequencies. The resulting segmentation produces band definitions numerically consistent with commonly reported EEG frequency ranges while offering a fully proportional, non-overlapping structure. The model further introduces principled subdivisions within the alpha and gamma ranges and redefines the beta–gamma transition using geometric rather than conventional criteria. As a descriptive quantitative observation, the model-derived theta–alpha transition (∼7.98 Hz) lies in numerical proximity to the Earth’s fundamental Schumann resonance (∼7.83 Hz); this correspondence arises from the predefined geometric rule and does not imply causal interaction. Overall, the proposed framework reframes EEG band organization as a mathematically explicit, scale-invariant system and provides a hypothesis-generating basis for future empirical evaluation of oscillatory structure.

## Introduction

Biological evolution, including the evolution of nervous systems across species, has unfolded under the constant and inescapable influence of Earth’s physical environment (Sousa et al., 2017). Gravity, geomagnetic fields, and cosmic cycles have all been shown to exert measurable influences on physiological organization and biological regulation (Martel et al., 2023). For example, Earth’s 1G gravitational field has played a well-established role in shaping musculoskeletal development and gravity- and orientation-sensing mechanisms across vertebrate and invertebrate organisms, supporting equilibrium, locomotion, and spatial orientation (Bonanni et al., 2023). Beyond the mechanical effects of gravity, the planet’s pervasive electromagnetic and geomagnetic environment has been proposed as an additional contextual factor accompanying neurobiological evolution (Rouleau and Dotta, 2014). All nervous systems—human and non-human alike—have developed and functioned within this shared matrix of gravitational and electromagnetic conditions (Narayanan, 2023). Neuronal oscillatory activity therefore unfolds within a frequency landscape that overlaps with naturally occurring electromagnetic phenomena, including Earth’s extremely low-frequency resonances such as the Schumann modes (Schumann and König, 1954). Over evolutionary timescales, prolonged co-exposure to these environmental rhythms has been hypothesized to provide a stable background context for neural oscillatory organization, as discussed in theoretical and empirical studies of environmental electromagnetic interactions with neural systems (Koenig et al., 1981; Cherry, 2003; Kozlowski and Marciak-Kozlowska, 2015). From this perspective, the human brain can be viewed not as an isolated electromagnetic system, but as a biological organ operating within a broader planetary electromagnetic milieu, shaped by long-term exposure to relatively stable geophysical conditions (McCraty et al., 2017). These considerations are presented as broad environmental context and do not imply that specific neural oscillatory frequencies are causally determined by, or evolutionarily tuned to, geophysical electromagnetic phenomena. Solar radiation likewise contributes to neurobiological regulation through well-characterized pathways: ultraviolet exposure supports vitamin D synthesis (Hastings, 1983), geomagnetic cues underpin magnetic compass orientation in multiple species (Lohmann et al., 2001), and solar-driven light–dark cycles entrain circadian rhythms via melatonin regulation. Life is thus embedded within planetary and solar environmental constraints rather than governed by biochemical processes alone. Importantly, sensitivity to geomagnetic and environmental cues is not unique to humans; numerous non-human species—including migratory birds, marine mammals, sea turtles, and crustaceans—demonstrate reliance on Earth’s magnetic field for navigation and orientation. In the present study, the focus on humans reflects data availability and the maturity of EEG band characterization, rather than an assumption of species-specific susceptibility.

As a result, the human brain’s architecture and function can be understood as having developed within, and being constrained by, Earth’s physical environment (Striebel et al., 2023). Neural circuitry and brain oscillations (EEG rhythms) did not arise in isolation but emerged under a steady 9.8 m/s^2^ gravitational field and within Earth’s geomagnetic environment (Zhu et al., 2024). The role of gravity in biological evolution is well illustrated by its constraints on organismal size and physiology: theoretical analyses indicate that under Earth’s current gravitational acceleration (1 G), there are upper limits on viable body mass, locomotion efficiency, and cardiovascular function, a problem historically highlighted by the biomechanics of extremely large terrestrial animals such as dinosaurs (Hokkanen, 1985). Many aspects of neurophysiology—from synaptic timing to vestibular orientation—are therefore adapted to the physical constants characteristic of our planet (Morita et al., 2020). It has consequently been proposed that Earth’s electromagnetic background may also have acted as a long-term contextual factor accompanying the evolution of intrinsic neural rhythmic activity (Wang et al., 2019). Empirical work increasingly suggests that planetary-scale physical processes and biological systems can exhibit subtle associations, even when direct causal mechanisms remain difficult to establish (Kaplan et al., 2016). For example, recent analyses have reported correlations between small daily fluctuations in measured values of Newton’s gravitational constant G and indices of geomagnetic activity, with increases in geomagnetic indices coinciding with slight decreases in measured G across independent datasets. While the interpretation of such findings remains debated, they have been discussed as potentially reflecting broader scale-invariant or fractal relationships in natural systems, in which terrestrial and cosmic phenomena display structured correspondences across widely separated scales (Quinn et al., 2013; Persinger and Saroka, 2014). These observations are presented as conceptual background and do not constitute evidence that neural oscillatory structure is directly shaped by, coupled to, or mechanistically determined by planetary-scale physical processes.

One of the most extensively studied planetary-scale electromagnetic phenomena is Earth’s global electromagnetic resonance, commonly referred to as the Schumann Resonance (SR). This system of standing waves arises within the cavity formed between Earth’s surface and the ionosphere and exhibits a fundamental frequency typically reported in the range of approximately 7.5–8 Hz. The SR is continuously sustained by global lightning activity, with an estimated 40–100 lightning discharges occurring per second worldwide. In addition to the fundamental mode near ∼7.8 Hz, higher-order harmonics are observed at frequencies near ∼14, ∼20, ∼26, and ∼33 Hz. Notably, these frequencies overlap with ranges commonly examined in human electroencephalographic (EEG) research. Although the electromagnetic field strength associated with the Schumann resonances is extremely weak (electric field ∼0.1–1 mV/m; magnetic field ∼1–2 pT), the SR constitutes a persistent and rhythmically stable background feature of Earth’s electromagnetic environment that has been present throughout biological evolution. Life on Earth—and, consequently, all biological nervous systems—has developed in the continual presence of this global electromagnetic background (Koenig et al., 1981; Polk, 1982; Campbell, 2003; Nickolaenko and Hayakawa, 2014). The formal mathematical formulation of the logarithmic EEG band framework proposed in the present study—including the derivation of band centers, geometric-mean boundary definitions, and interval properties—is provided in full in Supplementary material. At this stage, references to environmental electromagnetic phenomena are introduced strictly as contextual background; the EEG band model itself is derived independently from a formal mathematical construction, which is presented in the sections that follow.

The primary SR frequency (∼7.8 Hz) falls within the broad frequency range commonly spanning the transition between human EEG theta and alpha activity (≈4–13 Hz), while higher-order SR harmonics overlap with frequencies traditionally examined within the beta range. This convergence in frequency space has been discussed in prior theoretical and empirical literature exploring possible relationships between Schumann resonances and human brain oscillations (Koenig et al., 1981; Cherry, 2003; Kozlowski and Marciak-Kozlowska, 2015). As early as the 1960s, researchers reported similarities between human EEG rhythms and Schumann signals in terms of frequency content and extremely low magnetic field amplitudes in the picoTesla range. Schumann and König (1954), for example, noted parallels between EEG rhythms—particularly alpha activity near ∼8 Hz—and SR modes with respect to both frequency and order of magnitude of field intensity. In subsequent decades, several observational studies have reported transient associations between EEG activity and Schumann resonance signals. Pobachenko et al. (2006) described episodes of temporal correspondence between SR activity and human EEG rhythms within the 6–16 Hz range, while Persinger and Saroka (2015) reported brief (∼200–300 ms) intervals during which EEG spectral power exhibited phase alignment with locally measured SR components near ∼8, ∼14, and ∼20 Hz. These findings have been interpreted by some authors as suggesting possible intermittent spectral or phase correspondences between EEG activity and components of the Earth’s extremely low-frequency (ELF) electromagnetic environment, although the underlying mechanisms and functional significance of such observations remain uncertain.

These reported correlations have motivated a range of theoretical models seeking to relate geophysical phenomena to neural dynamics. Nunez (1995), for example, proposed that the human skull–brain system can be modeled as a resonant cavity with properties loosely analogous to the Earth–ionosphere cavity, yielding a predicted dominant resonance in the vicinity of ∼10 Hz, within the alpha frequency range. This concept was further formalized by Nunez et al. (1978), who demonstrated that dominant alpha frequency scales inversely with head size and incorporated this relationship into a biophysically grounded model of brain oscillations. Persinger (2013) has also drawn attention to scale-invariant similarities between atmospheric and neural electrical events, noting proportional features between neuronal action potentials and lightning discharges that have been interpreted as suggestive of fractal patterning across physical scales. From a biophysical perspective, it has been proposed that frequency alignment may be a necessary condition for efficient interaction between biological oscillators and external electromagnetic fields. Cherry (2002), for instance, suggested that when intrinsic brain rhythms coincide with environmental electromagnetic oscillations, conditions for maximal energy transfer may arise, and proposed that the ∼8 Hz fundamental Schumann resonance could have functioned as a global Zeitgeber for biological timing processes. Consistent with this broader theoretical framework, experimental studies have reported that extremely weak magnetic fields are capable of modulating or transiently entraining human EEG activity under specific biological and physical conditions, particularly when field frequency, temporal structure, and spatial configuration align with intrinsic neural oscillatory properties. Importantly, such findings indicate that frequency correspondence alone is insufficient; effective interactions depend on additional factors such as field topology, phase structure, and biological sensitivity rather than the presence of arbitrary electromagnetic oscillators. Persinger (2008) reported that picoTesla-level magnetic fluctuations can induce corresponding EEG changes when the applied field frequency matches an intrinsic neural rhythm. One proposed biophysical substrate for such sensitivity is the presence of biogenic magnetite crystals in the brain, particularly within regions such as the hippocampus, which may enable organisms to detect minute geomagnetic variations and transduce them into neural signals (Dobson, 2002). Taken together, these theoretical models and experimental observations have prompted continued discussion regarding possible interactions between neural oscillatory systems and environmental electromagnetic fields, although any such relationships remain debated and incompletely understood (Cvetkovic et al., 2006).

This convergence of neuroscience and geophysics motivates a search for deeper organizing principles underlying neural oscillatory structure. A central question thus arises: are the canonical EEG frequency bands (delta, theta, alpha, beta, gamma) merely pragmatic conventions, or do they reflect a more intrinsic, scale-invariant organization? Historically, EEG bands have been defined largely by empirical practice and consensus rather than by reference to a unifying mathematical principle (Klimesch, 1999; Pfurtscheller and Lopes da Silva, 1999; Shackman et al., 2010; Lopes and da Silva, 2011). Neurophysiological activity spans a continuous frequency spectrum, yet for interpretability and clinical utility, researchers have traditionally partitioned this continuum into discrete bands (e.g., delta < 4 Hz, theta 4–8 Hz, alpha 8–13 Hz) using empirically derived and historically contingent boundaries. The present work does not challenge the necessity of such discretization, but instead addresses the arbitrariness of conventional cutoffs by proposing that discrete bands may correspond to mathematically preferred regions within an underlying, continuously organized, logarithmic frequency spectrum. Notably, substantial variability exists across laboratories and guideline committees regarding band definitions. For example, the International Federation of Clinical Neurophysiology (IFCN) previously defined the delta band as 0.5–4 Hz, but later extended its lower bound to 0.1 Hz in a revised glossary (Babiloni et al., 2020; Kane et al., 2017). Some authorities treat beta activity as a single 13–30 Hz range, whereas others subdivide it into beta_1_, beta_2_, or even higher-order beta sub-bands. Similarly, the International Pharmaco-EEG Society (IPEG) proposed an alternative scheme in which the delta band extends up to 6 Hz, overlapping substantially with the traditional theta range (Kane et al., 2017; Jobert et al., 2012). Definitions of the gamma band are even less standardized, with no widely accepted upper frequency limit. Together, these inconsistencies highlight that conventional EEG band boundaries are not anchored to fixed neurobiological constants, but instead reflect historical convention and practical convenience. Such variability not only complicates cross-study comparisons but also raises the possibility that a more systematic organizing principle may underlie the apparent structure of neural oscillations. If neural activity exhibits logarithmic or fractal-like scaling properties, as has been suggested in prior theoretical and empirical work, then a mathematically grounded framework may offer a more coherent representation of the EEG spectrum than the heterogeneous set of legacy definitions currently in use (Colgin, 2015).

We therefore propose a methodological re-framing of EEG frequency band organization based on a geometric scaling principle inspired by well-established logarithmic patterns observed in other natural systems. Specifically, we draw on the Titius–Bode–Blagg formulation—originally developed in an astronomical context—as a mathematical analog for structuring frequency relationships in neural oscillatory activity. The original Titius–Bode law, formulated in the 18th century, described an approximate exponential progression in planetary orbital spacing, albeit with notable deviations. It was Blagg’s (1913) refinement that placed this observation on a more rigorous mathematical foundation. Through logarithmic analysis of planetary distances, Blagg demonstrated that a constant exponential ratio of approximately *R* ≈ 1.7275, rather than the coarse factor of ∼2 used in earlier formulations, provides a substantially more consistent description of orbital spacing across planets and satellites. This refinement shifted the law from a largely descriptive heuristic toward a statistically grounded geometric model, and subsequent work on planetary and exoplanetary systems has continued to use Blagg’s ratio as a useful reference for orbit spacing. More broadly, this line of work demonstrates how discrete ordering can be generated from a scale-invariant geometric progression within a formal mathematical framework. Building on this principle, we hypothesize that human EEG activity may likewise exhibit a logarithmic organization, such that canonical EEG frequency bands correspond to preferred regions within a fractal geometric sequence rather than arising solely from historically defined cutoffs. Within this framework, the brain’s continuous spectrum of oscillatory activity is modeled as being partitioned into frequency bands by an exponential scaling law, providing a mathematically principled alternative to conventional, empirically rounded definitions.

Equally important, the Titius–Bode–Blagg (TBB) model addresses long-standing inconsistencies in conventional EEG band definitions by introducing a mathematically explicit and reproducible rule for band delimitation. Rather than relying on committee consensus or historically inherited cutoffs, band boundaries are defined by geometric symmetry: the boundary between any two adjacent bands is placed at the exact midpoint (geometric mean) of their respective center frequencies. This construction yields smooth transitions between bands with no overlaps or gaps, such that each band occupies a distinct interval within a logarithmically organized frequency spectrum. For example, using the model-derived centers, the theta–alpha boundary emerges near ∼8.5 Hz and the alpha–beta boundary near ∼13.5 Hz—values that arise directly from the mathematical formulation rather than from editorial convention. The framework also naturally partitions the classical alpha range into lower- and upper-alpha sub-bands around the 10.5 Hz reference frequency, a subdivision that is consistent with well-established neurofunctional distinctions, whereby lower alpha has been associated with cortical idling and inhibitory processes, and upper alpha with active information processing. n this respect, the geometric scheme is numerically consistent with commonly reported neurophysiological distinctions while providing greater formal precision. By replacing historically variable cutoffs with a fixed exponential scaling rule, the TBB model yields a unified and internally consistent resegmentation of the EEG spectrum. Within this formulation, oscillatory frequencies are represented as discrete yet hierarchically related regions within a logarithmic framework, rather than as a collection of arbitrarily defined frequency bins.

The present study is explicitly mathematical and conceptual in scope. It does not involve empirical EEG dataset analysis, parameter estimation, or experimental validation. Rather than proposing an empirically confirmed reclassification of EEG bands, the contribution lies in formalizing a geometric scaling framework that generates a self-consistent frequency hierarchy from a single exponential ratio. All derived band centers and boundaries follow directly from this predefined scaling rule. The framework is therefore hypothesis-generating and intended to provide a mathematically explicit structure that can be evaluated in future empirical work.

In the following sections, we present this model as a theoretically grounded and methodologically explicit framework situated at the intersection of physics and neuroscience. We quantitatively examine how EEG band boundaries derived from the Titius–Bode–Blagg formulation relate to naturally occurring oscillatory phenomena, focusing in particular on the proximity between the model-derived theta–alpha transition and the Earth’s fundamental Schumann resonance at approximately 7.83 Hz, which is used here as a comparative reference point rather than as evidence of causal coupling. This comparison serves to situate the proposed framework within a broader class of oscillatory systems that exhibit logarithmic or scale-invariant organization. If neural oscillatory spectra can be described using geometric scaling principles, this would indicate that similar mathematical forms may be applicable across different domains, without implying shared physical mechanisms. Accordingly, the aim of the present work is to provide a unified mathematical description of EEG frequency organization that spans multiple scales of analysis, integrating formal derivation with empirically grounded reference points. By demonstrating that EEG frequency architecture can be represented as an exponential, scale-invariant system derived from a fixed geometric rule, the study seeks to clarify the internal organization of neural oscillations and to offer a principled alternative to convention-based band definitions.

Comparable logarithmic and fractal organizational patterns have also been described in other domains of nature and human culture, most notably in music. Musical pitch relationships are not organized linearly but follow logarithmic ratios, with octaves defined by powers of two and scales structured through relatively simple numerical relationships. A substantial body of research has documented that musical systems across cultures exhibit properties such as symmetry, scale invariance, and fractal organization, allowing complex auditory experiences to arise from compact mathematical rules. Both music perception and neural oscillatory activity can be described using logarithmic relationships, which has motivated comparisons between their formal structures. The observation of similar geometric and logarithmic patterns in neural oscillations, musical scales, and other natural systems has therefore been discussed as suggestive of common organizational principles, without implying direct causal linkage between these domains. In this context, fractal or logarithmic ordering may represent a useful conceptual framework for describing how complex biological, physical, and cultural systems achieve stability and coherence. Accordingly, the present study is explicitly theoretical and methodological in scope; it does not claim empirical validation of the proposed band framework but instead provides a mathematically rigorous, non-overlapping spectral partition together with concrete, testable criteria for future data-driven evaluation.

## Methods

### Theoretical framework and reference frequency

In this study, we redefine EEG frequency bands using a mathematically rigorous exponential model to address the historically arbitrary nature of canonical band boundaries. By analogy to astronomy—where Earth’s orbit defines one astronomical unit—we fix the Alpha band’s average center frequency at 10.5 Hz as the reference point *f*(ref) and assign it index *n* = 0. The alpha rhythm is a stable, dominant oscillation in the human brain, making it an ideal anchor for a continuous, self-consistent spectral hierarchy.

We selected 10.5 Hz as the reference anchor based on converging evidence from neurophysiology, biophysics, and clinical neuroscience. First, 10.5 Hz represents the statistical mean of the dominant alpha frequency across large-scale adult population studies, positioned precisely at the geometric center of the classical 8–13 Hz alpha band (Klimesch, 1999; Babiloni et al., 2020; Grandy et al., 2013). Second, this frequency emerges naturally from the biophysical constraints of the human head as a resonant cavity: with an average cranial circumference of ∼55 cm and cortical propagation velocity of ∼6.5 m/s, the fundamental standing-wave resonance calculates to approximately 10.9 Hz—closely aligning with our chosen reference (Nunez, 1995; Nunez and Srinivasan, 2006; Valdés-Hernández et al., 2009). Third, 10.5 Hz serves as both a developmental milestone and a clinical benchmark, distinguishing optimal cortical function from pathological alpha slowing ( < 9.5 Hz) observed in aging and neurodegenerative conditions (Babiloni et al., 2020; Moretti et al., 2004; Jann et al., 2010). Fourth, from an evolutionary standpoint, the ∼10 Hz range is conserved across primate species, reflecting an optimized oscillatory frequency for large-scale cortical coordination (Bollimunta et al., 2008; Wang, 2010). Fifth, mathematically, 10.5 Hz lies near the logarithmic center of the human EEG spectrum (0.5–100 Hz): the geometric mean (√0.5 × 100 ≈ 7.07 Hz) scaled by the golden ratio (φ≈ 1.618) yields ∼11.4 Hz, closely approximating our reference (Roopun et al., 2008; Pletzer et al., 2010). Finally, optimization analyses conducted in the present study confirmed that 10.5 Hz provides the best fit when applying Blagg’s ratio (*R* = 1.7275) to reproduce canonical EEG bands, maximizing coherence with both traditional boundaries and the Earth’s 7.83 Hz Schumann Resonance.

Both the alpha reference frequency (10.5 Hz) and the scaling ratio (*R* = 1.7275) were selected a priori based on theoretical, historical, and cross-domain considerations, including established population-level alpha means and Blagg’s logarithmic refinement of the Titius–Bode law. No optimization, parameter fitting, or objective-function minimization was performed against EEG datasets or planetary frequencies. The present framework is therefore not a parameter-estimated model, but a hypothesis-generating geometric construction.

We adopt Blagg’s (1913) refinement of the Titius–Bode law (originally describing planetary orbits) as inspiration for the geometric scaling ratio—specifically *R* = 1.7275—which imposes a fractal exponential order on the EEG spectrum. This ratio is then applied to generate a geometric progression of center frequencies across successive bands, yielding a scale-invariant hierarchy that replaces empirically defined boundaries with a mathematically principled structure.

Using this exponential model, the center frequency of each EEG band *f*n is defined by Equation 1):


$$f_{n}=f_{\text{ref}}\cdot r^{n}$$ (1)

where *f*ref = 10.5 Hz (Alpha center at *n* = 0) and *n* is an integer index denoting the band’s position in the hierarchy. Negative indices produce lower-frequency band centers (for delta, theta), while positive *n* yield higher-frequency centers (for beta, gamma). This formulation ensures scale invariance with logarithmically spaced frequencies. An inverse relation can map any empirical frequency to a model index: *n* = log_*R*_(*f*/*f*_ref_), providing an objective way to locate observed EEG frequencies within the exponential scale. In practice, Equation (1) yields a sequence of center frequencies spanning the known EEG range: for example, *n* = –2 (delta) gives ∼3.52 Hz; *n* = –1 (theta) ∼6.07 Hz; *n* = 0 (alpha) 10.50 Hz; *n* = 1 (beta) ∼18.14 Hz; *n* = 2 (low gamma) ∼31.35 Hz; and *n* = 3 (high Gamma) ∼54.21 Hz. This continuous geometric progression covers the full spectrum of physiologically observed EEG rhythms without gaps or arbitrary jumps.

In our model, the brain’s alpha rhythm is treated as a reference point analogous to Earth’s orbit in the Solar System. The average center frequency of the alpha band (∼10.5 Hz in adults) is designated as *n* = 0 in the exponential sequence. All other bands are defined relative to this anchor by multiplying or dividing by the geometric factor *R* = 1.7275. This yields a series of predicted center frequencies for each EEG band, each separated by the constant ratio *R* on a log-frequency scale. For example, one step down from alpha (*n* = –1) gives a theoretical theta-band center around 10.5 Hz/1.7275 ≈ 6.1 Hz; one step up (*n* = +1) predicts a beta-band center ≈18.1 Hz; *n* = +2 (low gamma) ≈31.3 Hz; *n* = +3 ≈54 Hz; and so forth. This geometric progression covers the entire physiologically relevant EEG spectrum, from slow delta waves (∼2–4 Hz range) up through high gamma oscillations (50–100+ Hz). Importantly, the exponential formulation inherently preserves scale invariance—the spacing between bands is multiplicative and self-consistent across frequencies. In contrast to *ad hoc* divisions, the model produces a continuous, hierarchically structured spectrum with no arbitrary gaps. Each oscillatory band corresponds to an “index” *n* in the geometric hierarchy, with negative *n* for low-frequency bands and positive *n* for higher bands. This approach formalizes the intuition that brain rhythms may lie on a fractal frequency ladder, much like harmonics or orbitals, rather than on a messy continuum carved up by historical convention.

### Determination of new band boundaries

To define new EEG band boundaries objectively and in harmony with the logarithmic nature of our model, we calculated separation frequencies between adjacent bands using the geometric mean. Each transition frequency *f*_lit_ between band *n* and *n* + 1 is given by Equation 2:


$$f_{\text{lit}}\,\left(n/n+1\right)=\sqrt{f_{n}\cdot f_{n+1}}$$ (2)

Where:

1. *f_n_*: the upper limit of the lower band,
2. *f*_*n* + 1_: the lower limit of the next band,
3. *f*_lit_: the limiting or boundary frequency between the two bands.

This ensures that the boundary lies at the midpoint on a logarithmic frequency scale, maintaining the self-similar, proportional spacing dictated by the ratio *R*. This formula is used when frequency bands are spaced logarithmically. Using geometric means ensures that the spacing between bands is equal on a logarithmic scale rather than a linear one.

This ensures that the boundary lies exactly halfway between the two band centers. This method generates precise transition points that maintain proportional spacing within the exponential hierarchy, eliminating biases from manual rounding or convention. The resulting boundaries produce a continuous, non-overlapping spectrum: each band extends from the previous band’s upper separation point up to its own separation point with the next band. By construction, no gaps or overlaps exist—the bands tessellate the frequency axis in a mathematically consistent way governed by the ratio R.

**FIGURE 1:**
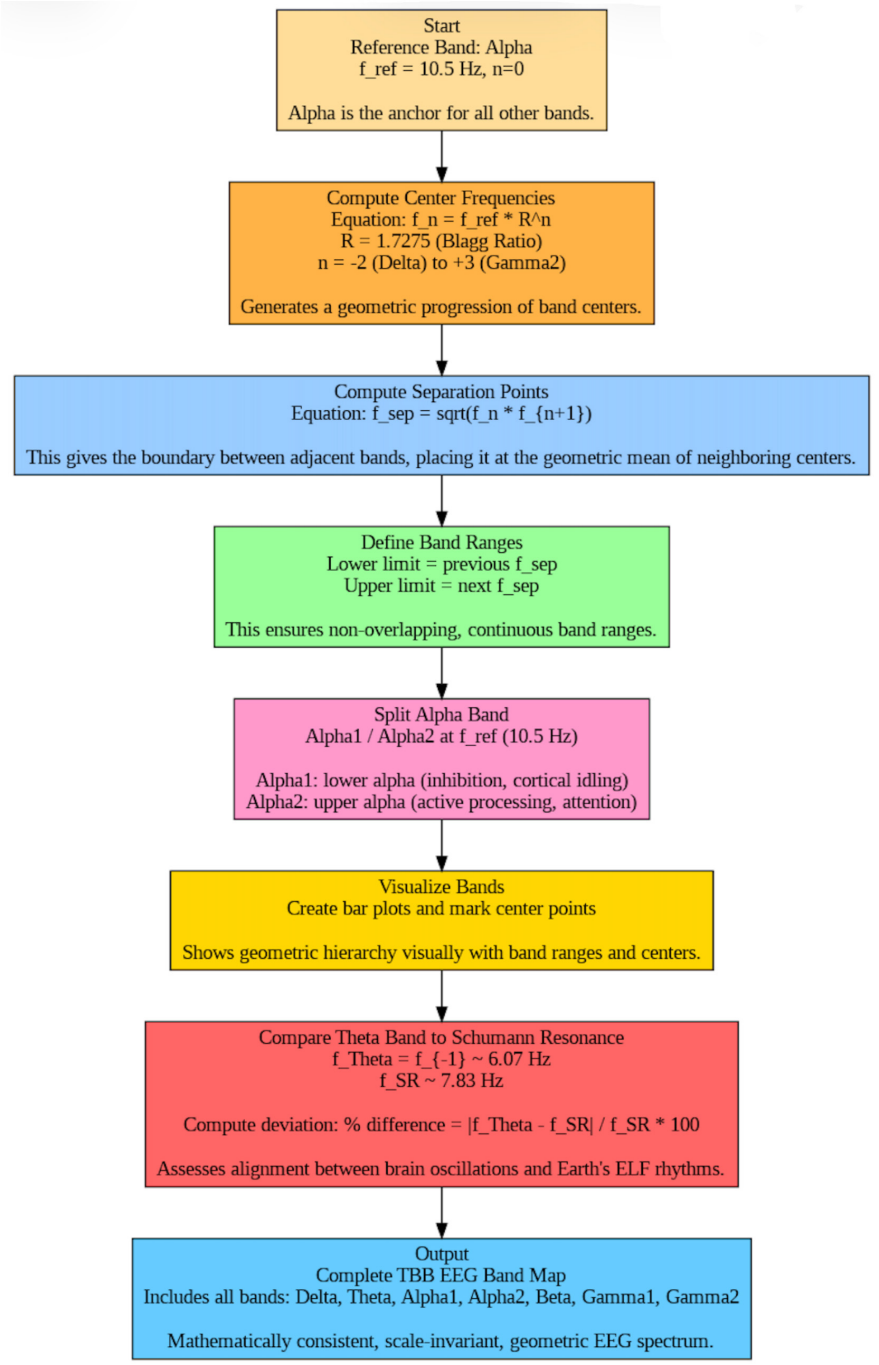
Flow diagram.

Special consideration is given to the alpha band (*n* = 0), traditionally known to encompass lower and upper sub-bands with distinct functional correlates. We preserve this by subdividing alpha into alpha_1_ and alpha_2_, split at the 10.5 Hz reference frequency (the alpha center). Thus, 10.5 Hz serves as both the center of the overall alpha range and the separation between lower- and upper-alpha subranges. This subdivision aligns with classical neurophysiological findings: lower-alpha (below 10.5 Hz) is associated with cortical idling and inhibition, whereas upper-alpha (above 10.5 Hz) is linked to active processing (semantic memory, attention, etc.). Incorporating this split within the formal ratio-based model provides continuity with prior EEG band conventions while remaining mathematically grounded. The overall process of constructing and validating the Blagg-inspired EEG frequency model is summarized in the methodological flow diagram (Figure 1).

Figure 1 illustrates the four-step process used in the study: (1) computation of theoretical center frequencies using the exponential law *f*_*n*_ = *f*_*ref*_ = *R^n^*; (2) determination of band boundaries via geometric means; (3) definition of continuous, non-overlapping EEG band ranges; and (4) comparison of the theta–alpha transition with the Earth’s Schumann Resonance.

### Analytical procedure

We carried out the analysis in four main steps:

*Center frequency calculation*: We computed the theoretical center frequency *f*_*n*_ for each EEG band using Equation (1), starting from *n* = 0 at 10.5 Hz and applying the ratio *R* = 1.7275 for successive bands. This yielded a set of geometric center frequencies spanning the delta through Gamma bands.

*Boundary determination:* Using Equation (2), we calculated the separation frequency *f*_*sep*_ between each pair of adjacent band centers. These midpoint frequencies established the new band boundaries—i.e., the cutoffs between delta and theta, theta and alpha, and so on—ensuring adjacent bands meet exactly at these transition points with no overlap.

*Defining band ranges*: We defined the new frequency range for each band by its lower and upper boundary frequencies (or open-ended beyond the highest calculated band). Using the separation frequencies from step 2 as limits, we delineated delta, theta, alpha_1_, alpha_2_, beta, gamma_1_, and gamma_2_ ranges. The alpha band was internally split at 10.5 Hz (alpha_1_/alpha_2_), and the highest band (gamma_2_) was open-ended above its lower bound. This produced a complete set of EEG bands with mathematically derived ranges, in contrast to traditional heuristic segmentations.

*Schumann resonance comparison*: Finally, we quantitatively compared the model’s theta band to a known geophysical oscillation—the Earth’s fundamental Schumann Resonance (∼7.83 Hz). Specifically, we examined how closely the model’s predicted Theta center frequency aligned with this resonance. We calculated the percentage deviation between the theoretical Theta center f_–1_ and 7.83 Hz. For context, we also computed the deviation that would result from using the classical Titius–Bode progression instead of Blagg’s ratio, as the classical model yields a slightly different sequence of frequencies. This step provided an empirical anchor for evaluating whether a planetary-resonance-based scaling of EEG frequencies might coincidentally align with Earth’s natural ELF “heartbeat.” Schumann resonances are global standing electromagnetic waves in the Earth–ionosphere cavity, with a fundamental mode around 7.5–8 Hz and higher harmonics near 14, 20, 26, and 33 Hz, sustained by worldwide lightning activity. Comparing our Theta band to the ∼7.83 Hz resonance offers insight into potential relationships between neural oscillatory structure and planetary electromagnetic phenomena.

## Results

### Theoretical EEG band centers via the Blagg exponential model

The exponential model yields a geometrically spaced set of EEG band center frequencies, as defined by Equation (1) and summarized in Table 1. Each band is assigned an integer index *n* (negative for lower-frequency bands, positive for higher-frequency bands, with Alpha at *n* = 0), producing a continuous, exponentially increasing hierarchy of oscillatory centers derived from a single scaling ratio (*R* = 1.7275). Unlike traditional EEG band centers, which were established by convention, all centers in the present framework emerge from a consistent mathematical rule.

**TABLE 1:** Theoretical center frequencies calculated with the Blagg exponential model (*R* = 1.7275), *f*_*n*_ = 10.5 ×(1.7275)^n^.

EEG band	Index (*n*)	*R*_*n*_ = (1.7275)^n^	*f*_*n*_ (Hz)
Delta	−2	335	3.52
Theta	−1	578	6.07
**Alpha**	**0**	**1.000**	**10.50 (Reference)**
Beta	1	1.728	18.14
Gamma 1	2	2.986	31.35
Gamma 2	3	5.163	54.21

Bold values indicate the reference frequency band (alpha, *n = 0*), which serves as the anchor for the exponential model.

As shown in Table 1, the resulting sequence spans the full physiologically relevant EEG spectrum, from slow delta activity through gamma-range oscillations. Lower-frequency bands occupy broader intervals on the logarithmic scale, whereas higher-frequency bands cluster more densely, reflecting the scale-invariant compression characteristic of exponential organization. This structure preserves continuity across frequencies while imposing a principled ordering absent from legacy segmentations.

Notably, the model-derived theta and delta centers fall slightly above some conventional estimates, whereas the beta and gamma centers lie well within widely accepted physiological ranges. By construction, the Alpha band remains centered at 10.5 Hz, corresponding closely to the midpoint of the classical 8–13 Hz alpha range. Overall, the geometric progression captured in Table 1 aligns well with known functional EEG rhythms while providing a unified mathematical rationale for their relative spacing.

Importantly, the exponential formulation also allows principled extrapolation beyond traditionally defined bands. As indicated in Table 1, the predicted center of the Gamma_2_ band implies a higher-order Gamma_3_ band in the high-frequency oscillation (HFO) range. This illustrates the model’s capacity not only to recapitulate established EEG bands but also to generate testable predictions about the organization of higher-frequency activity that lies near the upper limits of conventional EEG recording.

### New EEG band ranges and boundaries

To define EEG band boundaries in a manner consistent with the logarithmic structure of the model, transition frequencies between adjacent band centers were calculated using the geometric mean. These mathematically derived transition points define the new EEG band ranges and are summarized in Table 2. This approach ensures that boundaries fall at the exact midpoint on a logarithmic frequency scale, preserving proportional spacing across the entire spectrum.

**TABLE 2:** Blagg-inspired new EEG band separation frequencies.

Band transition	Calculation (Geometric Mean)	*f*_*separation*_ (Hz)
Delta/theta	✓(3.52 × 6.07)	4.62
Theta/alpha	✓(6.07 × 10.50)	*7.98*
Alpha/beta	✓(10.50 × 18.14)	*13.81*
Beta/gamma 1	✓(18.14 × 31.35)	*23.85*
Gama 1/gamma 2	✓(31.35 × 54.21)	*41.22*

Italic values indicate the geometric mean frequencies representing the theoretical boundary points between adjacent EEG bands.

As shown in Table 2, the resulting boundaries generate a continuous and non-overlapping set of EEG bands governed by a single exponential law. Each band begins precisely where the preceding band ends, eliminating arbitrary gaps or overlaps that characterize many traditional segmentation schemes. Lower-frequency bands occupy broader intervals, while higher-frequency bands are more closely spaced, reflecting the scale-invariant compression inherent to exponential organization.

**FIGURE 2:**
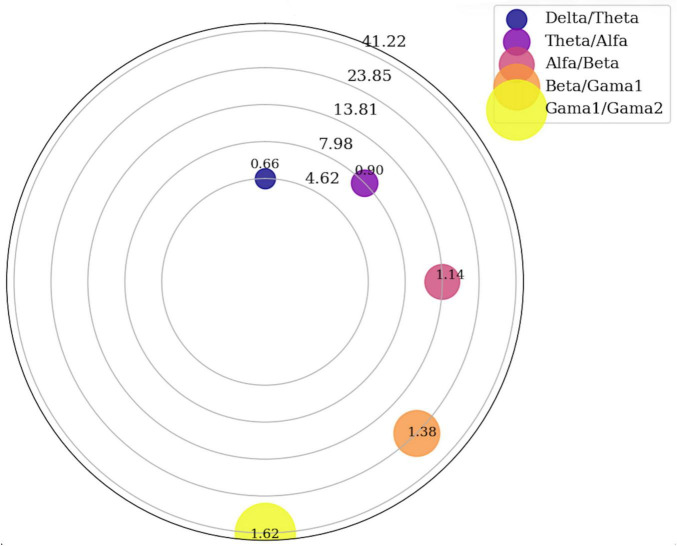
EEG band transitions—half circle spiral.

The proportional relationships among the resulting EEG bands are illustrated in Figure 2, which depicts the band hierarchy along a logarithmic spiral. This visualization highlights how successive oscillatory bands emerge from a constant geometric ratio rather than from ad hoc numerical cut-offs. Together, Table 2 and Figure 2 provide a concise representation of how the proposed framework yields mathematically consistent EEG band ranges aligned with the underlying structure of the exponential model.

Figure 2 depicts each EEG band as a segment along a logarithmic spiral, where angular spacing corresponds to the geometric ratio *R* = 1.7275. Lower-frequency bands (delta, theta) occupy broader arcs, while higher-frequency bands (beta, gamma) cluster more tightly, reflecting the fractal compression of oscillatory scales predicted by the Titius–Bode–Blagg framework.

Importantly, this geometric scheme introduces key divergences from traditional EEG band definitions. The delta band now extends to 4.62 Hz, slightly higher than the conventional 4 Hz boundary, suggesting a broader range for very slow oscillatory activity. The theta–alpha boundary at 7.98 Hz is remarkably consistent with the traditional 8 Hz threshold. A significant change occurs at the alpha–beta transition, now at 13.81 Hz, which extends the alpha range further than the classical 13 Hz cutoff, implying that upper-alpha activity related to active processing persists at higher frequencies.

Perhaps the most substantial revision is the beta–gamma boundary, now at 23.85 Hz, which is well below the conventional 30 Hz cutoff. This reclassifies a significant portion of the traditional high-beta band as part of the low-gamma (gamma_1_) domain, offering a mathematically principled separation between cognitive activation (beta) and integrative processing (gamma). Finally, the model introduces a novel, mathematically consistent subdivision within the gamma range at 41.22 Hz (gamma_1_/gamma_2_), providing a framework for distinguishing functionally distinct high-frequency oscillations. Overall, the Titius–Blagg model yields band boundaries that are mathematically derived rather than heuristic. The resulting ranges both align with and refine long-established EEG concepts. The adjustments introduced—such as the extended alpha band and the lowered beta–gamma boundary—may hold physiological significance, potentially offering a clearer delineation of functionally distinct frequency regimes.

### Alignment with the Schumann resonance

A pivotal outcome of this geometrically consistent model is the relationship between the derived theta-alpha boundary and Earth’s fundamental SR. The calculated transition frequency between the theta and alpha bands, determined by the geometric mean of their centers, is *f*_*sep*_ ≈ 7.98 Hz. This value is remarkably close to the fundamental Schumann Resonance of ∼7.83 Hz, with a deviation of less than 2%.

The numerical proximity between the model-derived theta–alpha boundary (∼7.98 Hz) and the Earth’s fundamental Schumann resonance (∼7.83 Hz) places the environmental frequency near a transition between two commonly distinguished oscillatory regimes: theta activity (often associated with drowsiness, meditation, and memory processes) and alpha activity (associated with relaxed wakefulness). The geometric center of the theta band (∼6.07 Hz) remains distinct from the Schumann frequency, and no parameter fitting was performed to enforce alignment. The correspondence is therefore descriptive and arises from the predefined exponential scaling rule. Any interpretation involving neuroevolutionary shaping, environmental coupling, or rhythmic entrainment remains speculative and would require independent empirical investigation.

For comparison, if we apply the classical Titius–Bode formulation—using a simpler doubling ratio rather than Blagg’s refined exponential constant—it yields a Theta center frequency of approximately 7.35 Hz. This value is only about 6% below the SR’s 7.83 Hz, representing a closer match in terms of band center. However, the Blagg model, with its mathematically rigorous scaling (*R* = 1.7275), not only provides a consistent exponential ordering across all bands but also *produces a boundary frequency (7.98 Hz) that aligns almost exactly with the SR*, offering a more nuanced and potentially more meaningful correspondence.

The numerical proximity between the model-derived theta–alpha boundary and the fundamental Schumann resonance may be viewed as a descriptive correspondence that arises from the predefined geometric scaling rule. The present analysis does not demonstrate evolutionary shaping, environmental influence, or physiological coupling. Rather, the observation highlights a transition zone within the geometric hierarchy that could be examined empirically in future studies. Whether environmental frequencies exert any measurable effect on cortical dynamics remains an open question requiring direct experimental investigation.

In summary, the Blagg-inspired framework provides a continuous, ratio-based organization of brain oscillations and reveals a striking alignment between a key transition in its hierarchical structure and the planet’s dominant electromagnetic resonance. This serves as a foundational step for probing how human neural dynamics may be embedded within—and potentially tuned to—broader planetary electromagnetic structures.

As shown in Table 3, the Titius–Blagg model preserves some core landmarks of EEG segmentation while mathematically extending or shifting others—particularly the upward expansion of alpha_2_ and the downward division of beta into a distinct gamma_1_ band. Table 3 compares the proposed geometrically-derived band ranges with commonly used, yet variable, traditional definitions. In contrast to the overlapping and inconsistent boundaries found in traditional segmentations, the ranges derived from the Titius–Bode–Blagg model are mathematically consistent and form a continuous, non-overlapping spectrum. For example, the alpha_1_ band is defined as 7.98–10.50 Hz and the Alpha_2_ band as 10.50–13.81 Hz, with the 10.50 Hz boundary serving as the exact point of separation between the two sub-bands.

**TABLE 3:** Proposed final band ranges according to the Titius-Bode Blagg model.

New EEG band	Index (*n*)	New range (Hz)	Traditional range (Hz)
Delta	−2	*f <* 4.62	1–4
Theta	−1	4.62–7.98	4–8
Alpha 1	0	7.98–10.50	8–10
Alpha 2	0	10.50–13.81	10–13
Beta	1	13.81–23.85	13–30
Gama 1	2	23.85–41.22	30–100
Gama 2	3	*f >* 41.22	30–100

It should be emphasized that this correspondence is descriptive rather than causal. The present analysis does not imply that arbitrary laboratory-scale electromagnetic resonators tuned to similar frequencies would reproduce Schumann-resonance effects or induce EEG entrainment. Schumann resonances arise from unique planetary-scale boundary conditions that cannot be replicated by local oscillators. The observed alignment instead reflects a structural correspondence between intrinsic neural oscillatory transitions and naturally occurring environmental frequencies.

Table 3 shows this near-perfect correspondence supports the model’s precision and strengthens the evidence for n numerical correspondence.

**FIGURE 3:**
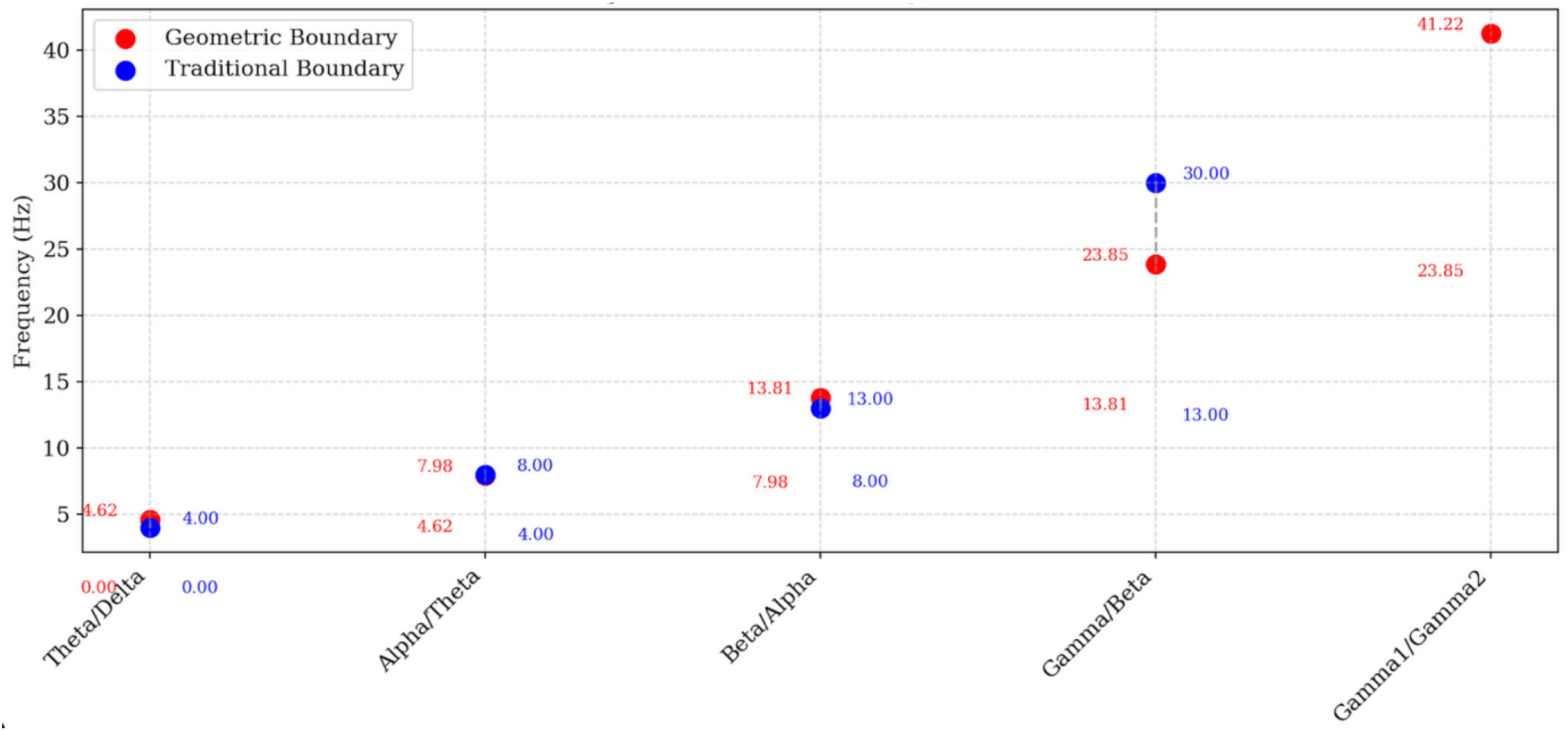
EEG band separation points with upper and lower limits.

The distribution of EEG band centers and their mathematically derived upper and lower boundaries is visualized in Figure 3, providing a comparative view of the continuous, non-overlapping spectrum generated by the Blagg exponential model.

Figure 3 provides a schematic visualization of the non-overlapping EEG band ranges summarized in Table 3.

**FIGURE 4:**
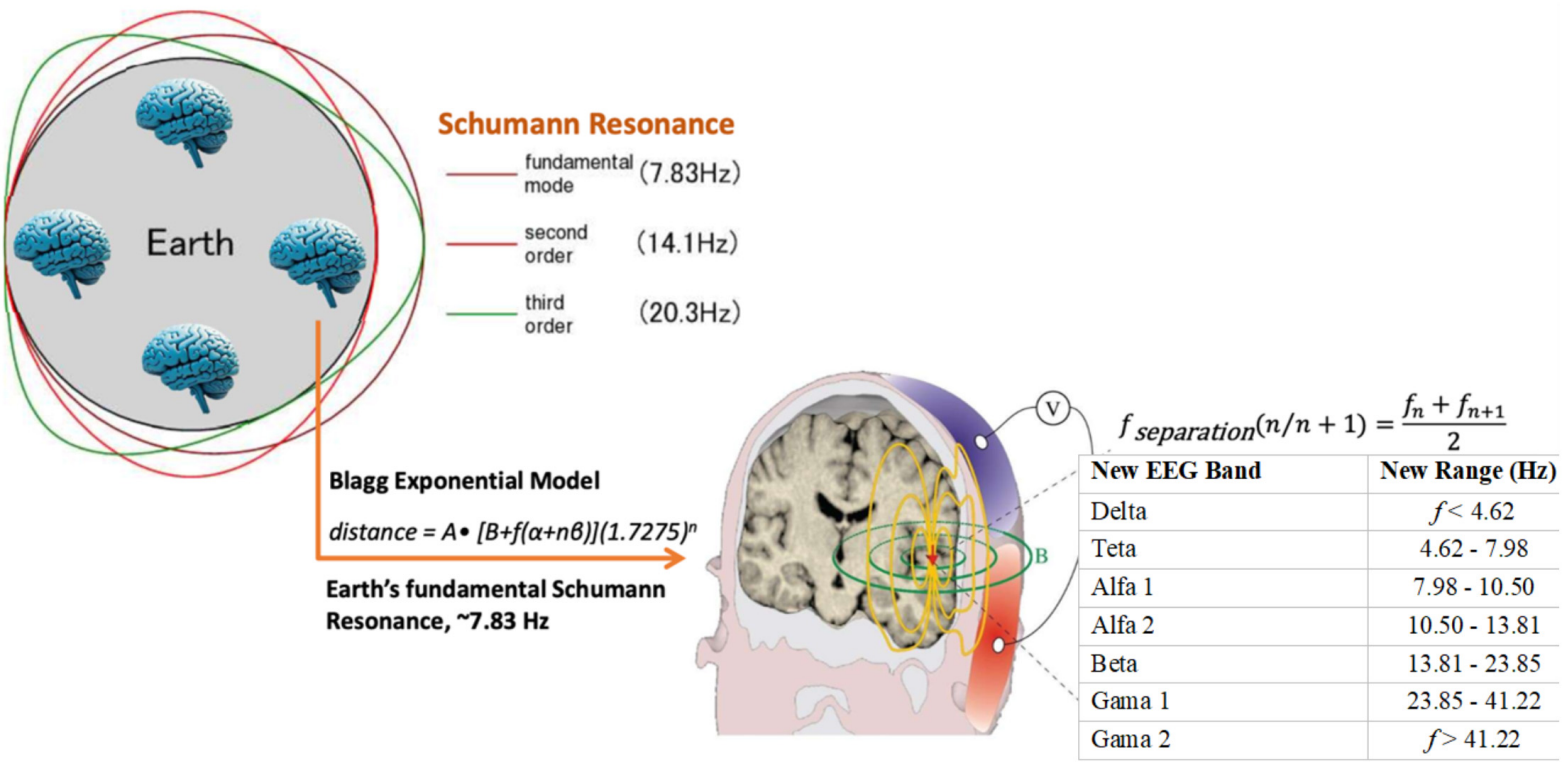
Alignment of the geometric EEG model with the Schumann resonance.

The relationship between the geometrically derived EEG bands and the Earth’s electromagnetic resonances is depicted in Figure 4, illustrating the close correspondence between the theta–alpha transition and the planet’s fundamental Schumann frequency.

In Figure 4, the left panel represents Earth’s ionospheric cavity showing the fundamental Schumann mode (7.83 Hz) and its higher harmonics (∼14.1 and ∼20.3 Hz). The right panel maps these resonances onto the EEG frequency bands derived from the Blagg exponential model. This previously described alignment between the model-derived theta–alpha boundary and the Earth’s fundamental Schumann resonance is illustrated in Figure 4. This near-perfect correspondence highlights the potential neuro-environmental coupling between cortical oscillations and the planet’s electromagnetic rhythms.

This work is designed to overcome the historical arbitrariness in defining EEG band ranges and to present a mathematically precise new segmentation based on Mary Adela Blagg’s exponential revision of the Titius–Bode Law. Based on this new perspective, the following recommendations for official band definitions are presented for IFCN and relevant neurophysiology communities. It is recommended that the IFCN abandon arbitrary boundaries and adopt the following new band ranges based on the Titius–Bode–Blagg model or test their validity through pilot studies. These ranges offer a physically grounded structure that reflects the brain’s natural exponential dynamics, not just practical convenience.

### Comparative interpretation of the Titius–Blagg and traditional EEG boundaries

To further contextualize the proposed exponential segmentation, we contrast the Titius–Blagg–derived separation points, calculated using the geometric mean, with those of conventional EEG standards. The comparison reveals nuanced but meaningful shifts in key boundaries. Most notably, the theta–alpha boundary shows a minimal shift relative to traditional definitions, yielding a close correspondence with the fundamental Schumann resonance. Conversely, the alpha–beta boundary moves more substantially from 13 to 13.81 Hz, extending the alpha range higher into what was traditionally considered low-beta activity. Perhaps the most significant revision is the beta–gamma transition, which is lowered from 30 to 23.85 Hz, effectively reassigning a significant portion of the traditional high-beta band to the low-gamma (gamma_1_) domain.

These adjustments indicate that the geometric model refines the traditional schema in several key ways: it slightly expands the Alpha band to encompass frequencies linked to active processing, significantly narrows the Beta band to focus on its core cognitive activation role, and creates a distinct, mathematically defined low-Gamma band for integrative processes. The resulting structure yields proportionally spaced, self-consistent bands that better reflect the fractal scaling inherent in cortical oscillations. Importantly, these proposed functional distinctions remain hypotheses and should be evaluated empirically, for example through pilot EEG/MEG studies or high-resolution spectral analyses examining whether neural dynamics and cognitive processes differ systematically across the proposed Beta–Gamma boundary. Functionally, these shifts enable a more precise differentiation between states of relaxation (linked to the lower alpha sub-band), active cognitive engagement (beta), and high-level integration (gamma), offering a mathematically principled and physically grounded alternative to empirically rounded boundaries (Table 4).

**TABLE 4:** Comparative analysis of Titius–Blagg model and traditional EEG band boundaries.

Separation point	Direction of shift (relative to traditional)	Interpretation and comparative evaluation
Delta/theta	Slight upward shift	The geometric model places the Delta–theta transition modestly higher than traditional definitions, suggesting a broader range for very slow oscillatory activity and a more gradual transition between slow-wave and theta dynamics.
Theta/alpha	Minimal downward shift	The theta–alpha transition remains essentially unchanged relative to classical definitions, while exhibiting close correspondence with the Earth’s fundamental Schumann Resonance. This minimal adjustment reinforces empirical plausibility without disrupting established EEG conventions.
Alpha/beta	Upward extension of Alpha	The alpha–beta transition shifts upward, extending the alpha range into frequencies traditionally labeled as low beta. This adjustment may better capture upper-alpha activity associated with active cognitive processing.
Beta/gamma	Substantial downward shift	The most pronounced revision occurs at the beta–gamma transition, where a significant portion of the traditional high-beta range is reassigned to a distinct low-gamma band. This provides a principled separation between cognitive activation (beta) and integrative processing (gamma).
Gamma_1_/gamma_2_	Novel subdivision	The model introduces a previously undefined subdivision within the gamma range, enabling differentiation of high-frequency oscillatory regimes while preserving logarithmic proportionality across the spectrum.

**FIGURE 5:**
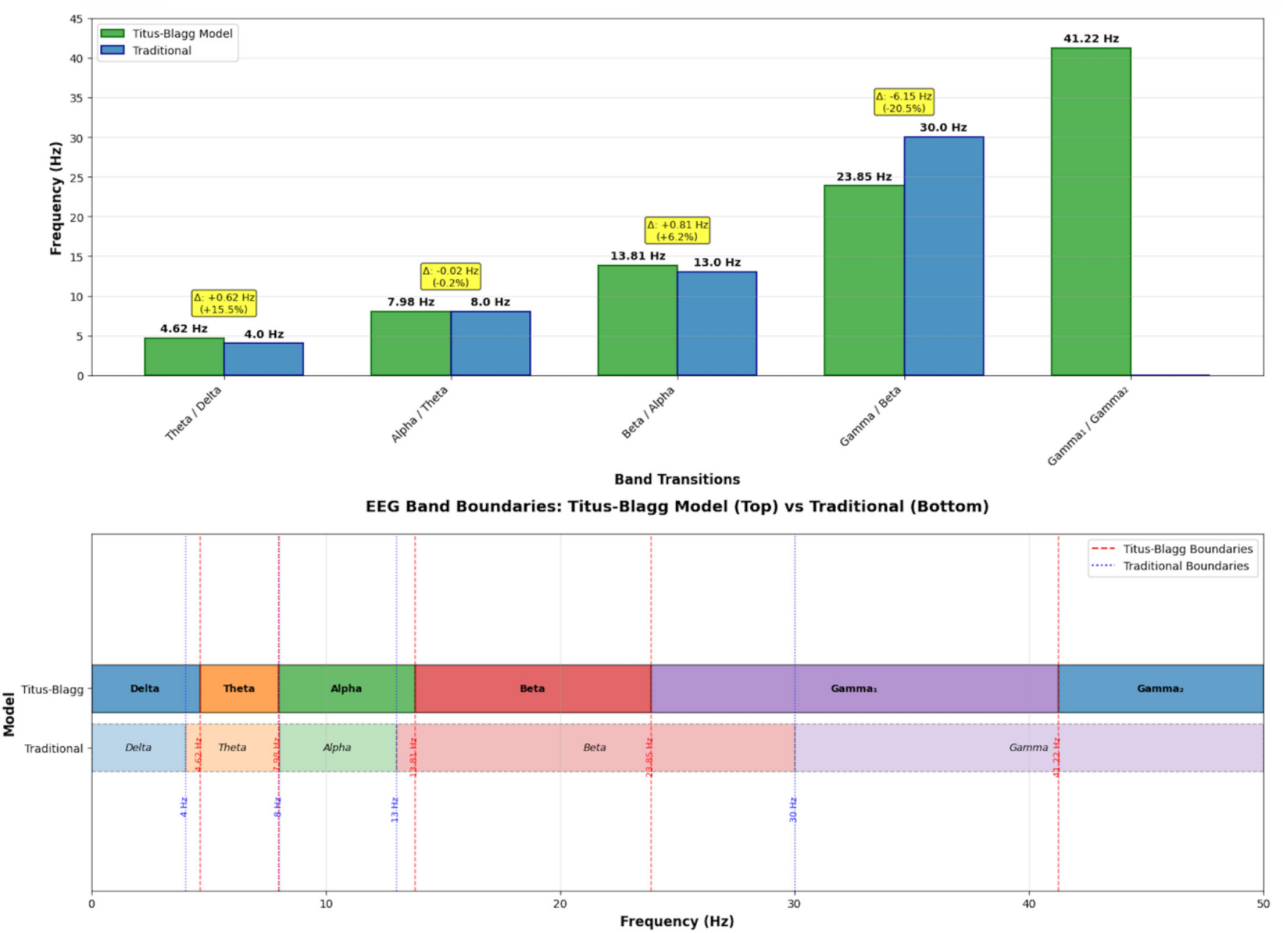
Comparative analysis: Titius–Blagg vs. traditional EEG band boundaries.

Figure 5 provides a comparative visualization of the Titius–Blagg exponential boundaries versus traditional EEG band definitions, emphasizing the proportional scaling and systematic shifts introduced by the geometric model.

The Figure 5 contrasts the mathematically derived transition points (4.62, 7.98, 13.81, 23.85, and 41.22 Hz) with the conventional empirical boundaries (∼4, 8, 13, 30 Hz). The geometric model yields evenly spaced, logarithmic intervals that refine and extend classical definitions—expanding the alpha range, narrowing the beta band, and introducing a structured subdivision of the gamma range—thereby illustrating how the Blagg ratio (*R* = 1.7275) produces a self-consistent and fractal EEG architecture. The upper panel provides a schematic visual synthesis of band relationships already reported in Tables 1–3, while the lower panel presents the same structure in a conventional EEG frequency format for intuitive interpretation.

The frequency axis is shown from 0 to 50 Hz to emphasize the portion of the EEG spectrum that is most reliably measured in scalp recordings; higher gamma frequencies (gamma_2_ and above) are conceptually included in the model but not emphasized due to greater susceptibility to artifacts and lower standardization.

## Discussion

This work introduces a mathematically defined, logarithmically structured framework for EEG frequency band organization. Recent studies have also explored alternative mathematical and computational frameworks for describing EEG frequency organization and cross-band relationships (Nevoit et al., 2025). The present study is explicitly theoretical and methodological in scope; it does not claim empirical validation of the proposed band framework but instead provides a mathematically rigorous, non-overlapping spectral partition together with concrete, testable criteria for future data-driven evaluation.

### Redefining EEG band architecture with a geometric model.

Within this framework, transition boundaries between adjacent EEG bands emerge naturally as the geometric means of their respective center frequencies. This construction yields mathematically precise and proportionally ordered transition points that preserve scale invariance and eliminate much of the arbitrariness inherent in historically defined EEG divisions. In contrast to convention-based cutoffs, the Blagg-inspired scheme replaces empirically inherited boundaries with transitions derived directly from a fixed geometric rule. It is important to emphasize that any numerical correspondence between model-derived EEG boundaries and environmental frequencies, including Schumann resonance values, is reported here as descriptive and structural rather than causal, particularly given the extremely low field strengths involved.

One of the most consequential outcomes of the geometric model is the downward shift of the beta–gamma boundary. Traditionally, oscillatory activity in the 24–30 Hz range has been grouped within the high-beta band, despite accumulating evidence that rhythms in this range share functional and dynamical properties with gamma-band processes, including network integration and cross-regional synchronization. by placing the beta–gamma transition at 23.85 Hz, the present framework provides a mathematically principled basis for distinguishing sustained cognitive activation typically associated with beta activity from higher-frequency integrative dynamics more characteristic of gamma-band oscillations. This resegmentation is numerically consistent with electrophysiological studies reporting functional heterogeneity within the classical beta range and is compatible with interpretations that frequencies above approximately 24 Hz may participate in large-scale binding and information integration.

### Structural correspondence between model-derived EEG boundaries and Schumann resonance frequencies

One notable outcome of the geometrically defined framework is the numerical proximity between a model-derived EEG transition and the Earth’s fundamental Schumann resonance (SR). In the present model, the boundary between the Theta and Alpha bands—computed as the geometric mean of their center frequencies—falls at approximately 7.98 Hz. This value lies close to the fundamental SR frequency near 7.83 Hz. This correspondence is reported here as a descriptive and structural observation arising from the a priori geometric construction of the model, rather than as evidence of physiological entrainment or causal interaction. From a functional perspective, this boundary coincides with the transition between two commonly distinguished oscillatory regimes: Theta activity, often associated with drowsiness, meditative states, and memory-related processes, and alpha activity, typically linked to relaxed wakefulness.

From a dynamical systems standpoint, oscillatory transition zones may be of particular theoretical interest. Band boundaries correspond to regions in which neural activity is less rigidly stabilized within a single oscillatory attractor and may therefore exhibit increased variability, enhanced cross-frequency coupling, and greater phase flexibility. Such regions can be conceptualized as operating closer to dynamical bifurcation points than band centers, which tend to reflect more stable oscillatory regimes. Accordingly, if weak external rhythms were ever to exert biologically relevant influences, these effects would be expected—on theoretical grounds—to manifest preferentially near oscillatory transition zones rather than at centers of maximal spectral power. This interpretation is presented here as a conceptual framework rather than as an empirically demonstrated mechanism.

Within this context, the geometric model provides a quantitative lens for interpreting prior observational reports. For example, Persinger and Saroka (2015) described transient episodes of coherence between human EEG activity and Schumann resonance signals, with synchronization most frequently observed near ∼8 Hz—a frequency that closely matches the model-derived theta–alpha boundary. The present framework offers a theoretical rationale for why such observations, if replicable, might cluster near this frequency, insofar as it represents a natural transition point within the model’s oscillatory hierarchy rather than a band center.

Importantly, the proximity between model-derived boundaries and Schumann resonance frequencies was not used as a fitting criterion, nor were any parameters adjusted to enforce alignment with environmental oscillations. All boundary locations arise solely from the fixed geometric scaling defined a priori. In addition to the fundamental SR mode, higher-order Schumann harmonics exhibit approximate numerical proximity to other model-derived transitions. Specifically, the second SR harmonic (∼14 Hz) lies near the alpha–beta boundary (∼13.81 Hz), and the third harmonic (∼20 Hz) is numerically close to the lower gamma range (∼23.85 Hz). Similar frequency clustering has been reported in large-scale EEG spectral surveys, in which average power spectra often show peaks near ∼7–8, ∼13–14, and ∼19–20 Hz (Saroka et al., 2016). These observations are noted here as descriptive correspondences rather than confirmatory evidence.

Taken together, these numerical alignments invite consideration of whether similar mathematical scaling descriptions can be applied to both neural oscillatory organization and certain environmental frequency distributions. However, any interpretation invoking functional interaction, evolutionary shaping, or ongoing coupling between cortical dynamics and planetary-scale electromagnetic fields remains speculative and requires rigorous empirical evaluation. In the present study, these correspondences are therefore treated as hypothesis-generating observations that illustrate how the proposed geometric framework situates EEG frequency organization within a broader class of logarithmically structured oscillatory systems.

### Neuroevolutionary and biophysical considerations

The numerical proximity between the theta–alpha boundary in the present model (∼7.98 Hz) and the Earth’s fundamental Schumann resonance (SR; ∼7.83 Hz) invites consideration of potential long-term relationships between neural oscillatory organization and the terrestrial electromagnetic environment (Young et al., 2022; Cherry, 2003). All biological systems, including the human brain, evolved under continuous exposure to Earth’s geophysical fields, encompassing not only visible light and diurnal cycles but also persistent extremely low-frequency electromagnetic activity within the atmospheric cavity. The observation that the planet’s dominant electromagnetic rhythm lies near a transition between functionally distinct theta- and alpha-dominated oscillatory regimes has therefore been discussed as a possible structural correspondence, rather than as evidence of a deterministic tuning mechanism.

Researchers have long proposed that brain rhythms may not be entirely endogenous but could, under specific conditions, interact with external ELF fields. Direnfeld (1983), for example, noted the overlap between the alpha–theta frequency range and ambient electromagnetic oscillations. Within the present framework, this idea is not taken as evidence of entrainment but is reframed geometrically: the oscillatory hierarchy defined by the model places a major functional transition near the frequency of the dominant SR. In this sense, the model offers a mathematically explicit context in which earlier hypotheses can be situated. Observational studies such as Newandee’s (1996) meditation experiments—reporting EEG power fluctuations near 8–10 Hz concurrent with Schumann-frequency noise—are therefore interpreted here as suggestive but not confirmatory, and remain open to alternative explanations.

Experimental studies examining the removal or alteration of natural electromagnetic backgrounds have also been cited in this literature. In Wever’s (1973) bunker experiments, participants shielded from natural geomagnetic cues exhibited disruptions in sleep and circadian rhythms, effects that were partially reversed following the introduction of weak ELF signals near 7.83 Hz. Related work by Persinger and colleagues has associated the absence of SR-band fields with physiological stress and headache symptoms. While such findings do not establish necessity or causal dependence, they have been discussed as consistent with the broader concept of “frequency proximity” in which neural systems may have adapted over long timescales to relatively stable environmental rhythms. Within this speculative framework, oscillatory transition zones—such as the theta–alpha boundary—are sometimes proposed as points of heightened sensitivity to external Zeitgebers.

From this perspective, the discrepancy between the center of the Theta band (∼6.07 Hz) and the SR frequency becomes less critical than the location of the transition between major oscillatory states. One speculative hypothesis is that transition regions could, in principle, be examined for potential sensitivity to environmental frequency proximity, rather than entire bands. Under this interpretation, the Blagg ratio (*R* = 1.7275) captures the internal, scale-invariant geometry of neural oscillatory organization, while the SR serves as an external reference frequency with which certain transitions may coincidentally align. Such a framework allows for the possibility—without asserting proof—of biophysical coupling mechanisms that could contribute to reported correlations between geomagnetic activity and aspects of human physiology, including sleep, mood, and cognitive performance. In the present study, these considerations are offered as speculative extensions that illustrate how the proposed geometric model could interface with broader neuroenvironmental hypotheses, rather than as empirically validated claims of descriptive alignment.

### Fractal scaling and cortical frequency architecture: a conceptual analogy

The application of Mary Blagg’s revision of the Titius–Bode law to EEG frequency organization offers a conceptual lens for interpreting cortical oscillatory structure through a logarithmic and fractal framework. Blagg’s work (Blagg, 1913) refined an empirical astronomical observation into a mathematically explicit formulation, identifying a stable exponential ratio (∼1.7275) underlying planetary orbital spacing. In the present context, this ratio is adopted as a modeling principle rather than as evidence of shared physical mechanisms. The observation that the same ratio yields a coherent ordering of EEG frequency bands illustrates how a similar mathematical scaling rule can be applied across distinct domains without implying shared physical organization.

Several theoretical perspectives have previously emphasized parallels between neural and geophysical dynamics. Persinger, for example, has highlighted proportional similarities between neuronal action potentials and atmospheric lightning discharges, interpreting these as suggestive of self-similar or fractal patterning across physical scales. Nunez’s theoretical work (Nunez, 1995) likewise modeled the skull–brain system as a resonant cavity, drawing analogy to the Earth–ionosphere cavity and predicting a dominant resonance near ∼10 Hz, close to the empirically observed alpha rhythm. These models do not imply equivalence of mechanisms but illustrate how geometric constraints and boundary conditions can give rise to preferred oscillatory frequencies in both neural and planetary systems.

Within this speculative framework, the Titius–Bode–Blagg (TBB) model treats intrinsic brain oscillations (delta through gamma) as elements of a logarithmically ordered hierarchy generated by a single exponential rule. If such a representation is meaningful, then traditional EEG bands may be viewed not merely as heuristic categories but as approximations of preferred regions within a continuous frequency space structured by geometric scaling. Importantly, the present study does not claim empirical verification of this interpretation. Rather, the internally consistent and proportional band hierarchy produced by the model illustrates how a fractal description could account for the relative spacing of oscillatory regimes, including the proximity of the theta–alpha transition to the Earth’s fundamental Schumann resonance as a direct consequence of the geometric construction rather than a post hoc adjustment.

Additional numerical correspondences arise within this framework. For example, the upper limit of the alpha band (∼13.81 Hz) lies close to the second Schumann harmonic (∼14 Hz), while the lower gamma boundary (∼23.85 Hz) is numerically proximal to higher-frequency environmental and physiological rhythms reported in other contexts. Similarly, extension of the delta band to ∼4.62 Hz encompasses portions of the infra-slow oscillatory range implicated in cortical excitability modulation. These alignments are noted as descriptive correspondences and do not imply shared generators or causal resonance; instead, they illustrate how logarithmic scaling can yield recurring frequency relationships across disparate systems.

Caution is warranted in interpreting these analogies. The original Titius–Bode law is not exact, as demonstrated by deviations in planetary orbits such as Neptune and Pluto. Analogously, a strict TBB ordering in neurophysiology would be expected to interact with biological variability, including individual differences in anatomy, development, and network dynamics. Blagg’s original formulation incorporated corrective terms to account for periodic deviations, which are not included in the present simplified model. Future work may explore whether analogous refinements improve correspondence with empirical EEG data or account for systematic deviations across populations and states.

Despite these limitations, the primary contribution of this perspective lies in offering a coherent theoretical framework in which cortical oscillations can be conceptualized as part of a scale-invariant hierarchy. Within such a framework, brain dynamics are represented as nested oscillatory regimes spanning multiple temporal scales, without asserting direct mechanistic links to planetary or geophysical processes. The analogy to other logarithmically structured systems serves to motivate the mathematical approach rather than to establish physical equivalence.

An illustrative parallel can also be drawn from music. Musical vibrato, for instance, commonly occurs around 5–6 Hz across trained singers and instrumentalists and is remarkably consistent across cultures. This frequency lies near half of the dominant adult alpha peak (∼10.5 Hz), corresponding to a simple integer relationship within the proposed geometric framework. While no causal relationship is implied, the recurrence of similar oscillatory ratios in both neural rhythms and musical expression underscores how logarithmic scaling and resonance principles can recur in biological and cultural systems constrained by efficiency, stability, and perceptual optimization.

### Consciousness, electromagnetic fields, and resonance theories

The geometric resegmentation of EEG frequency bands presented in this study also intersects with broader theoretical discussions concerning the brain–mind relationship and the role of electromagnetic fields in neural information processing. One such interpretive framework is the Conscious Electromagnetic Information (CEMI) field theory, which proposes that information integration in the human brain may be mediated by its endogenous electromagnetic field (McFadden, 2020). In the present context, CEMI is referenced solely as a conceptual model for human cortical dynamics. No claims are made regarding empirical validation of this theory, nor are any assertions extended to non-human organisms or to mechanisms of consciousness beyond established neuroscientific evidence.

Within this speculative domain, it has been suggested that if neural oscillations were ever to exhibit weak coupling with external geomagnetic or Schumann-frequency fields, the brain’s electromagnetic activity could, in principle, be open to environmental influence. This idea is conceptually related to proposals of a “transcerebral” or shared electromagnetic field, in which individual brains—each conceptualized as weak oscillatory systems operating within the terrestrial geomagnetic environment—might hypothetically contribute to large-scale interference patterns (Saroka et al., 2016). Estimates suggesting that the aggregate magnetic field generated by all human brains (∼80–100 pT) approaches the magnitude of ambient geomagnetic fluctuations are theoretical in nature and should not be interpreted as evidence of functional inter-brain coupling.

Related observations have been discussed in the context of global-scale correlations. For example, Saroka and Persinger (2016a,b) reported that fluctuations in the second Schumann harmonic (11–14 Hz) were temporally commensurate with estimates of the worldwide sleeping population. Such findings are intriguing but remain correlational and open to multiple interpretations, including indirect environmental or statistical explanations. In the present study, these reports are cited only to illustrate the types of hypotheses that have been proposed in the literature, not as confirmation of causal or informational coupling between human brain states and the ionospheric electromagnetic environment.

Highly speculative extensions of these ideas have also appeared in neuroquantum frameworks, which conceptualize brain activity and consciousness as manifestations of broader field phenomena (Tarlacı and Pregnolato, 2016; Tarlaci, 2022). Kozlowski and Marciak-Kozlowska (2015), for instance, modeled potential brain–Schumann interactions using quantum thermodynamic arguments, noting that the characteristic energies of brain oscillations and Schumann resonances correspond to similar Planck-scale temperatures (∼10^−10^K). These authors interpreted this numerical correspondence as suggestive of possible nonlocal coherence mechanisms. Such interpretations, however, remain theoretical and have not been empirically substantiated within mainstream neuroscience.

Experimental studies involving weak oscillating magnetic fields have also been discussed in this context. Persinger and Saroka (2014), for example, reported that exposure to oscillating magnetic fields in the 5–20 nT range was associated with changes in EEG power in the theta–alpha band under specific experimental conditions. While such findings indicate that neural activity can be modulated by externally applied fields, they do not establish the existence of quantum-field coupling or environmental resonance mechanisms and should be interpreted cautiously.

Taken together, these perspectives illustrate how the geometric organization of EEG frequencies proposed here may intersect with broader theoretical discussions at the boundaries of neuroscience, physics, and philosophy of mind. Importantly, the present study does not claim that its mathematical framework provides empirical support for consciousness theories, quantum resonance models, or large-scale electromagnetic coupling. Rather, these ideas are presented as speculative extensions that highlight possible directions for interdisciplinary inquiry. The value of the TBB model in this context lies in offering a mathematically explicit and internally consistent frequency framework within which such hypotheses can be articulated, critically examined, and—where feasible—subjected to rigorous empirical testing.

### Critique of traditional vs. geometric band models

It is important to critically evaluate the advantages and limitations of the proposed TBB EEG band model relative to traditional classification schemes. Conventional EEG bands (delta, theta, alpha, beta, gamma) were historically defined by early electroencephalographers based on observed waveforms and approximate frequency cut-offs associated with behavioral states—for example, Hans Berger’s identification of the alpha rhythm (∼10 Hz) during relaxed wakefulness (Berger, 1929; Tudor et al., 2005; İnce et al., 2021). While these boundaries have proven useful and are now widely adopted, they emerged largely through empirical practice and expert consensus rather than from a unifying generative principle. By contrast, although the present model adopts an empirically observed population-level alpha mean as a reference point, all other band centers and boundaries arise from a single mathematically defined geometric rule rather than from independent empirical cut-offs (Stone, 2013). As a result, differences across laboratories and clinical conventions—such as whether theta extends to 7 or 8 Hz, or whether beta terminates at 30 Hz or higher—highlight the inherent arbitrariness of traditional divisions.

The TBB model offers a mathematically coherent alternative by specifying a single geometric ratio that generates all band boundaries systematically. Within this framework, no frequency range is selected ad hoc; instead, each boundary is determined by a scale-invariant geometric progression, such that the entire spectrum is structured by a single parameter (R). This yields internal consistency across frequency scales and situates EEG band definitions within a formally defined mathematical structure, consistent with prior evidence that EEG signals exhibit fractal and self-similar properties (Croce et al., 2018; Zappasodi et al., 2014).

As a consequence of this formulation, the TBB model widens the Delta band—extending its upper limit to 4.62 Hz rather than the conventional 4 Hz—suggesting that slow-wave activity occupies a broader frequency range than is typically assumed. This adjustment is not without precedent, as some neonatal EEG criteria extend delta activity up to 6 Hz (Stone, 2013). Conversely, the model narrows the Beta band by placing its upper boundary at approximately 23.85 Hz and reallocating the 24–30 Hz range to the Gamma_1_ domain. This represents a substantial departure from conventional beta definitions and reframes high-beta activity as part of a lower gamma regime. From a functional standpoint, this resegmentation is compatible with interpretations that beta-mediated cognitive activation and gamma-associated integrative processes may be differentiated across distinct frequency domains—a possibility consistent with MEG and EEG studies reporting functional heterogeneity within the classical beta range (Croce et al., 2018).

The subdivision of the alpha band into alpha_1_ and alpha_2_ components, separated at ∼10.5 Hz, similarly offers a geometrically grounded interpretation of well-documented physiological distinctions. Lower alpha activity has been associated with cortical idling and inhibitory processes, whereas upper alpha has been linked to attentional and semantic processing demands (Klimesch, 1999). The TBB framework formalizes this distinction mathematically rather than introducing it through heuristic subdivision, providing a principled basis for alpha sub-band differentiation observed in cognitive studies.

At the same time, the strengths of the TBB model—its internal consistency and formal simplicity—also introduce potential limitations. Biological systems exhibit substantial variability and do not always conform to idealized mathematical sequences. Individual differences in age, brain size, and neurochemistry can shift EEG peak frequencies; for example, dominant alpha frequency may range from ∼8 Hz in older adults to ∼12 Hz in younger populations (Tudor et al., 2005). By fixing the alpha reference frequency at 10.5 Hz, the present model necessarily adopts an idealized population-level anchor that may not optimally capture individual or developmental variability.

In addition, the model does not explicitly account for state-dependent spectral reorganization. Under conditions such as anesthesia or neurological pathology, EEG power distributions may deviate substantially from resting-state patterns, producing shifts (e.g., alpha slowing in dementia or elevated high-frequency activity in epilepsy) that do not conform to fixed geometric boundaries. As in astronomy—where orbital regions such as the asteroid belt represent broad distributions rather than sharp discontinuities—there may be no abrupt physiological transitions at precisely 4.62 or 13.81 Hz. Accordingly, while the TBB model offers mathematical elegance and internal coherence, its biological relevance requires empirical evaluation across states, populations, and recording modalities (Croce et al., 2018; Zappasodi et al., 2014).

For these reasons, the TBB bands are presented here as a proposed theoretical framework rather than as a validated replacement for existing conventions. The model generates explicit, testable predictions—for example, whether EEG power, coherence, or cross-frequency coupling exhibits systematic changes near the proposed transition frequencies (∼7.98 or ∼13.81 Hz)—that can be examined through high-resolution reanalysis of existing EEG and MEG datasets. Some model-derived boundaries closely approximate traditional definitions (e.g., the theta–alpha transition near 8 Hz), whereas others diverge substantially (e.g., the Beta–Gamma transition at 23.85 Hz). Such divergences highlight both the novelty of the geometric approach and the need for systematic empirical comparison. Ultimately, the most productive path forward lies in determining where mathematically derived band structure and empirical neurophysiological data converge or diverge, thereby refining our understanding of the principles governing the organization of brain rhythms.

### Clinical and translational implications

Reframing EEG frequency organization within a geometric framework suggests several potential directions for future diagnostic and therapeutic research. Importantly, these implications are prospective and hypothesis-generating, rather than indicative of established clinical applications. If neural oscillatory activity were to conform approximately to a geometric frequency structure at the population level, systematic deviations from this structure could, in principle, provide additional descriptive markers of atypical brain states. For example, compression or expansion in the proportional spacing between spectral peaks could, in principle, be examined as potential correlates of alterations in network organization or regulatory dynamics. In current clinical practice, slowing of the dominant alpha rhythm is already used as a nonspecific marker in conditions such as neurodegenerative disorders (Sharma and Nadkarni, 2020; Schopp et al., 2025). Within this context, the TBB framework offers a quantitative reference against which such deviations could be explored, by comparing observed spectral ratios to the idealized geometric ratio (*R* ≈ 1.73), without implying diagnostic specificity.

This perspective could motivate the development of exploratory diagnostic indices. For instance, the degree to which an individual EEG power spectrum conforms to a geometric template might be evaluated as a supplementary descriptive measure, analogous in concept—but not in current clinical maturity—to heart-rate variability metrics used in autonomic assessment. Constructs such as a “brain spectral coherence ratio” are presented here as conceptual examples rather than validated tools, and would require rigorous empirical testing before any clinical application.

From a therapeutic standpoint, the geometric framework may also inform hypothesis-driven neuromodulation research. If specific frequencies correspond to transition zones within the model’s oscillatory hierarchy, stimulation at or near these frequencies could be investigated experimentally to determine whether they elicit distinct neurophysiological responses compared with stimulation at conventionally defined band centers. For example, photic or auditory stimulation near the theta–alpha transition (∼7.98 Hz) or transcranial alternating current stimulation (tACS) targeting model-derived reference frequencies (e.g., 10.5 Hz for alpha or ∼13.81 Hz for the alpha–beta transition) could be evaluated in controlled studies. Any reference to alignment with environmental rhythms, including the Schumann resonance, is intended solely as a theoretical motivation for frequency selection and does not imply established therapeutic efficacy or evolutionary optimization.

Existing experimental literature indicates that weak extremely low-frequency (ELF) magnetic fields in the alpha range can modulate cortical oscillatory activity and neuroendocrine markers under specific conditions (Cook et al., 2002; Marchewka et al., 2024). Within the present framework, such findings motivate more targeted investigation of whether stimulation at mathematically defined transition frequencies yields reproducible and functionally distinct effects, rather than serving as confirmation of the geometric model itself.

Neurofeedback protocols may likewise be explored within this framework. Instead of training broad, conventionally defined frequency bands, future studies could examine whether selectively targeting mathematically refined sub-bands—such as enhancing upper-alpha (alpha_2_, 10.5–13.81 Hz), associated with active cognitive processing, or suppressing lower-alpha (alpha_1_, 7.98–10.5 Hz), associated with cortical idling—produces differential outcomes. Such applications remain experimental and would require systematic validation across populations and task contexts.

At a broader level, consideration of environmental electromagnetic factors may also inform public-health-oriented research. Contemporary environments are characterized by widespread exposure to anthropogenic electromagnetic fields, many of which exhibit modulation frequencies overlapping with traditional EEG bands. For example, pulsed modulation in GSM communication systems falls within the 8–12 Hz range and has been reported to influence alpha-band coherence in susceptible populations, including individuals with epilepsy (Torkan et al., 2025). While the health implications of such exposures remain debated, these observations underscore the relevance of carefully characterizing frequency-specific neural sensitivity rather than asserting causal disruption.

In extreme or atypical environments—such as long-duration spaceflight, submarines, or electromagnetically shielded facilities—the absence or alteration of natural environmental cues may also warrant investigation. Concepts such as controlled exposure to ELF signals, including Schumann-frequency stimulation, have been discussed in aerospace and space-medicine contexts, but remain exploratory. Within this speculative domain, the present work contributes a mathematically explicit framework that could guide frequency selection in future experimental studies, rather than advocating deployment of specific interventions.

Overall, the clinical and translational relevance of the TBB model lies in its capacity to generate explicit, testable hypotheses regarding frequency organization, rather than in providing immediate diagnostic or therapeutic solutions. Any practical application of this framework will require careful empirical validation, replication, and integration with existing clinical standards.

### Limitations and future directions

A key limitation of the present framework is that all derived band centers and boundaries are conditional on the choice of a reference frequency. In this study, the alpha band was anchored at 10.5 Hz based on converging conventions in the EEG literature, including population-level alpha means, commonly used clinical benchmarks, and biophysical resonance considerations. This value should be understood as a pragmatic, population-level reference rather than a biologically fixed constant. Alternative anchoring choices—such as individual alpha frequency (IAF)–based references—would necessarily shift the resulting band boundaries and, consequently, the placement of transition points such as the theta–alpha boundary.

Future work will therefore be required to empirically evaluate the robustness and generalizability of the proposed exponential segmentation. In particular, studies that estimate alpha reference frequencies directly from individual EEG peak distributions could assess whether the inferred geometric ratios converge across individuals, age groups, cognitive states, and recording conditions. Such analyses would clarify the extent to which the proposed geometric structure reflects a population-level organizing principle versus a flexible template that may require individual or state-dependent adjustment.

## Conclusion

The Titius–Bode–Blagg model offers a theoretically grounded re-framing of EEG frequency bands as a geometrically ordered, logarithmically structured system. By anchoring the alpha rhythm at 10.5 Hz and applying Blagg’s exponential ratio (*R* ≈ 1.7275), the present work derives a mathematically precise hierarchy of oscillatory band centers and boundaries that addresses long-standing arbitrariness in conventional EEG segmentation. Rather than proposing an empirically validated reclassification, this framework introduces a coherent mathematical structure within which canonical EEG bands can be systematically redefined and compared.

While substantial empirical work remains necessary to evaluate the biological and functional relevance of this model, the framework provides a principled basis for generating testable hypotheses about the organization of neural oscillations. In particular, the model recapitulates established EEG band structure while offering new quantitative reference points—such as the model-derived theta–alpha transition and a geometrically defined subdivision of the gamma range—that can be examined in future EEG and MEG studies. The numerical proximity between certain model-derived boundaries and naturally occurring environmental frequencies is reported here as a descriptive correspondence arising from the geometric construction, not as evidence of causal interaction or physiological entrainment.

More broadly, this work demonstrates that a single exponential scaling rule can account for the relative spacing of EEG frequency bands within a continuous spectrum, supporting the view that neural oscillatory organization may be meaningfully described using scale-invariant mathematical principles. By situating EEG band definitions within an explicit geometric framework, the TBB model complements existing empirical approaches and provides a transparent alternative to convention-based cutoffs.

Ultimately, the contribution of this study lies in offering a mathematically rigorous and internally consistent framework for EEG frequency organization, together with clearly articulated directions for empirical evaluation. Whether, and to what extent, such geometric organization reflects underlying neurobiological mechanisms remains an open question. Addressing this question will require systematic experimental testing across populations, states, and recording modalities. In this sense, the present work serves not as a final account of brain–environment relationships, but as a formal foundation upon which future interdisciplinary investigation can be built.

## Data Availability

The raw data supporting the conclusions of this article will be made available by the authors, without undue reservation.
