# Supplementary material for: Redefinition of EEG frequency bands: a fractal model inspired by Blagg’s Titius–Bode law
Source: Front Syst Neurosci. 2026 Mar 19;20:1736474. doi: 10.3389/fnsys.2026.1736474 (PMC13044072; doi:10.3389/fnsys.2026.1736474)
Supplement: Supplementary file 1 [file Supplementary_file_1.docx]

**Appendix A**

**Methodological Details:**

**Logarithmic Structuring of EEG Frequency Bands Using the Titius–Bode–Blagg (TBB) Model (R = 1.7275)**

**1. Introduction and Rationale**

This appendix provides a full methodological explanation of how EEG frequency bands were redefined using the **Titius–Bode–Blagg (TBB) geometric progression model**. The goal is to present the mathematical basis, the reasoning behind parameter selection, the computation of band centers and boundaries, and the practical implications of this logarithmic formulation. The section also aims to guide readers who may not be familiar with logarithmic or fractal frequency models, by connecting the mathematical structure to neurophysiological and biophysical reasoning. Traditionally, EEG frequency bands—Delta (1–4 Hz), Theta (4–8 Hz), Alpha (8–13 Hz), Beta (13–30 Hz), and Gamma (30–100 Hz)—have been defined empirically rather than mathematically. These boundaries vary slightly between studies, introducing ambiguity when comparing spectral analyses. The TBB model addresses this inconsistency by proposing that EEG bands follow a logarithmic (geometrically scaled) structure similar to that seen in other natural systems, including planetary orbits, musical intervals, and biological oscillations. In this framework, each successive frequency band center is obtained by multiplying the preceding one by a constant Blagg ratio (R). The model assumes that oscillatory systems in the brain are self-similar and scale-invariant, meaning that low and high frequencies share proportional relationships even if they operate on different timescales.

The TBB law was originally formulated to describe the geometric spacing of planetary orbits in the Solar System. Its broader implication is that many natural systems—astronomical, acoustic, and biological—exhibit *logarithmic ordering*. EEG oscillations, being rhythmic processes generated by coupled neuronal assemblies, also exhibit self-similarity across scales. EEG band boundaries were determined using a geometric progression derived from the TBB model, which assumes that oscillatory systems in nature follow logarithmic scaling. Instead of adopting traditional empirically fixed ranges (e.g., Delta 1–4 Hz, Theta 4–8 Hz), each band center was computed as:

$$f_{n}=f_{0}\times R^{n}$$

where $f_{0}=10.50\text{ }\text{Hz}\text{ }$represents the alpha-band center and the scaling ratio was fixed at R=1.7275. Boundaries between adjacent bands were defined by their geometric mean:

$$b_{n,n+1}=\sqrt{f_{n}f_{n+1}}$$

which produces equal spacing on a logarithmic frequency axis. All intervals were expressed in left-open, right-closed notation $(a,b]$, ensuring mathematically disjoint frequency ranges with no overlap between neighboring bands. This formulation yields the following corrected ranges:

| **Band** | **Range (Hz)** | **Center (Hz)** |
| --- | --- | --- |
| Delta | ≤ 0.00, 4.618] | 3.485 |
| Theta | (4.618, 7.988] | 6.078 |
| **Alpha₁** | (7.988, 10.500] | **9.159** |
| **Alpha₂** | (10.500, 13.801] | **12.038** |
| Beta | (13.801, 23.850] | 18.139 |
| Gamma₁ | (23.850, 41.220] | 31.340 |
| Gamma₂ | (41.220, 71.150] | 54.220 |

All EEG frequency bands are expressed using **half-open interval notation** $(a,b]$, which means:

- **a** is *excluded* from the interval
- **b** is *included* in the interval

Formally:

$$f\in\left( a,b \right] \text{if and only if}\text{ }a<f\leq b$$

This convention ensures that no frequency value belongs to more than one band. For example, $7.988\text{ Hz}$belongs to Theta, while $8.000\text{ Hz}$belongs to Alpha₁ — eliminating boundary overlap. The fixed ratio $R=1.7275$preserves internal logarithmic symmetry while keeping the Theta–Alpha transition (~7.988 Hz) close to the fundamental Schumann resonance (7.83 Hz), an alignment previously discussed as a possible biophysical correspondence. This model provides a mathematically consistent and non-overlapping framework for spectral analyses, facilitating reproducible comparisons across subjects and conditions. In this model, the Alpha band is divided into two sub-bands to better reflect functional differentiation:

- Alpha₁ (8–10.5 Hz) represents lower alpha activity, typically associated with relaxed attentional states and posterior rhythm synchronization.
- Alpha₂ (10.5–13.8 Hz) corresponds to upper alpha, often linked to semantic processing and cognitive control.

By defining these sub-bands geometrically rather than arbitrarily, the TBB framework aligns physiological interpretation with a consistent mathematical principle. The TBB partitioning thus defines disjoint yet harmonically nested intervals that support precise power, coherence, and causality analyses across frequency bands. This correction fully resolves the prior duplication error in the Alpha range and enforces geometric integrity across all spectral intervals.

**2. Mathematical Properties**

In logarithmic terms, the spacing between consecutive centers is uniform:

$$\log\left( f_{n+1} \right)-\log\left( f_{n} \right)=\log\left( R \right).$$

This property means that the EEG spectrum, when viewed on a logarithmic frequency axis, forms a perfectly symmetric and scale-invariant lattice. Each octave (in log f space) corresponds to a fixed multiplicative step of 1.7275. Because R > 1, higher-frequency bands widen progressively. This mirrors the neurophysiological reality that faster oscillations (beta/gamma) arise from smaller, faster, and more localized neural assemblies—thus occupying proportionally wider intervals in linear frequency but maintaining log-scale uniformity.

**3. Non-Overlapping (Disjoint) Definition**

One of the main methodological advantages of the Titius–Bode–Blagg (TBB) formulation lies in the way frequency bands are defined as mutually exclusive intervals. In traditional EEG literature, the boundaries between adjacent bands are usually given as approximate integers—e.g., “Theta = 4–8 Hz” and “Alpha = 8–13 Hz.” Although intuitive, this convention introduces an ambiguity: the frequency value exactly equal to 8 Hz may belong to both bands, depending on the researcher’s convention. Such overlaps, while numerically small, can propagate inconsistencies in spectral power calculations, coherence measures, and band-specific statistics. The TBB framework resolves this ambiguity by introducing a strict set-theoretic definition of frequency intervals on the real line. Each band $B_{n}$is defined as a half-open (left-open, right-closed) interval:

$$B_{n}=\left( \text{ }f_{\mathrm{sep}}\left( n-1,n \right),\text{ }f_{\mathrm{sep}}\left( n,n+1 \right) \right],$$

with the conventions

$B_{-2}=\left( -\infty,f_{\mathrm{sep}}\left( -2,-1 \right) \right]$for Delta and
$B_{+3}=\left( f_{\mathrm{sep}}\left( 3,4 \right),+\infty\right)$for the uppermost Gamma₂ band.

By construction, these intervals satisfy two key mathematical properties:

**Adjacency:**

$$f_{\mathrm{sep}}\left( n,n+1 \right)=\frac{f_{\mathrm{sep}}\left( n+1,n+2 \right)}{R},$$

meaning that the upper boundary of one band equals the lower boundary of the next when expressed on a logarithmic scale.

**Disjointness:** For any two distinct bands $B_{i}$and $B_{j}$with $i\neq j$,

$$B_{i}\cap B_{j}=\emptyset.$$

Hence, the entire positive frequency axis can be represented as the **disjoint union**

$$\bigcup_{n=-2}^{3} B_{n}=\left( 0,+\infty\right)$$

guaranteeing that every possible frequency belongs to exactly one band and never to two.

The choice of half-open intervals is not merely a notational convenience; it ensures determinism in numerical computations. In floating-point arithmetic, equality comparisons (e.g., f = 8.0) are sensitive to rounding error. If two adjacent bands were both defined with closed boundaries $\left[ a,b \right]$and $\left[ b,c \right]$, a frequency value that rounded to $b$could be assigned to *both*. Using $\left( a,b \right]$eliminates this ambiguity by systematically attributing the shared boundary $b$to the upper band only.

For instance:

- $f=7.980\text{ }\mathrm{Hz}$→ falls in Theta ($4.62<f\leq7.98$);
- $f=7.981\text{ }\mathrm{Hz}$→ immediately belongs to Alpha₁ ($7.98<f\leq10.50$).

Thus, even infinitesimal differences are resolved consistently, which is particularly important for automated spectral labeling or when iterating over thousands of discrete FFT bins. The formal definition can be expressed computationally as follows:

$$\text{Assign}(f)=\{\begin{matrix} \text{Delta}, & f\leq4.624473, \\ \text{Theta}, & 4.624473<f\leq7.988776, \\ \text{Alpha}_{1}, & 7.988776<f\leq10.500000, \\ \text{Alpha}_{2}, & 10.500000<f\leq13.800611, \\ \text{Beta}, & 13.800611<f\leq23.840556, \\ \text{Gamma}_{1}, & 23.840556<f\leq41.184561, \\ \text{Gamma}_{2}, & 41.184561<f\leq71.146329, \\ \text{High}, & f>71.146329. \end{matrix}$$

This conditional mapping can be implemented in any numerical environment (Python, MATLAB, R, etc.) without overlapping. When the *Power Spectral Density (PSD)* or coherence function is discretized (e.g., Δf = 0.5 Hz), each bin is guaranteed to map to exactly one band index. On a logarithmic axis, the band boundaries appear equally spaced because:

$$\log\left( f_{\mathrm{sep}}\left( n+1,n+2 \right) \right)-\log\left( f_{\mathrm{sep}}\left( n,n+1 \right) \right)=\log\left( R \right),$$

a constant value independent of n. These uniform spacing forms a lattice in log-frequency space, sometimes referred to as a “logarithmic tiling” of the spectrum. Each tile (band) is contiguous but non-overlapping, analogous to the way semitone intervals tile the audible frequency range in music theory. In contrast, conventional linear banding (e.g., equal-width 10 Hz bins) would create non-uniform ratios on the log scale, violating the self-similarity observed in biological oscillations.

Because every frequency map to one and only one band, statistical summaries such as mean band power, coherence, or Granger causality values are uniquely defined. In conventional definitions, spectral leakage or slight shifts in individual alpha frequency (IAF) can move energy across a fuzzy 8 Hz boundary, distorting comparisons. Here, even if individual peaks drift slightly, the classification remains deterministic. The (a,b] rule aligns with typical programming language conventions for array slicing—e.g., NumPy and MATLAB index intervals [start, end)—which simplifies implementation and avoids double counting. The set of all defined bands covers the positive frequency axis completely without gaps or overlaps:

$$\bigcup_{n=-2}^{3} B_{n}=\left( 0, 71.146329 \right] \text{and} B_{>3}=\left( 71.146329, \infty\right)$$

giving a continuous yet discretely segmented representation of EEG frequency space.


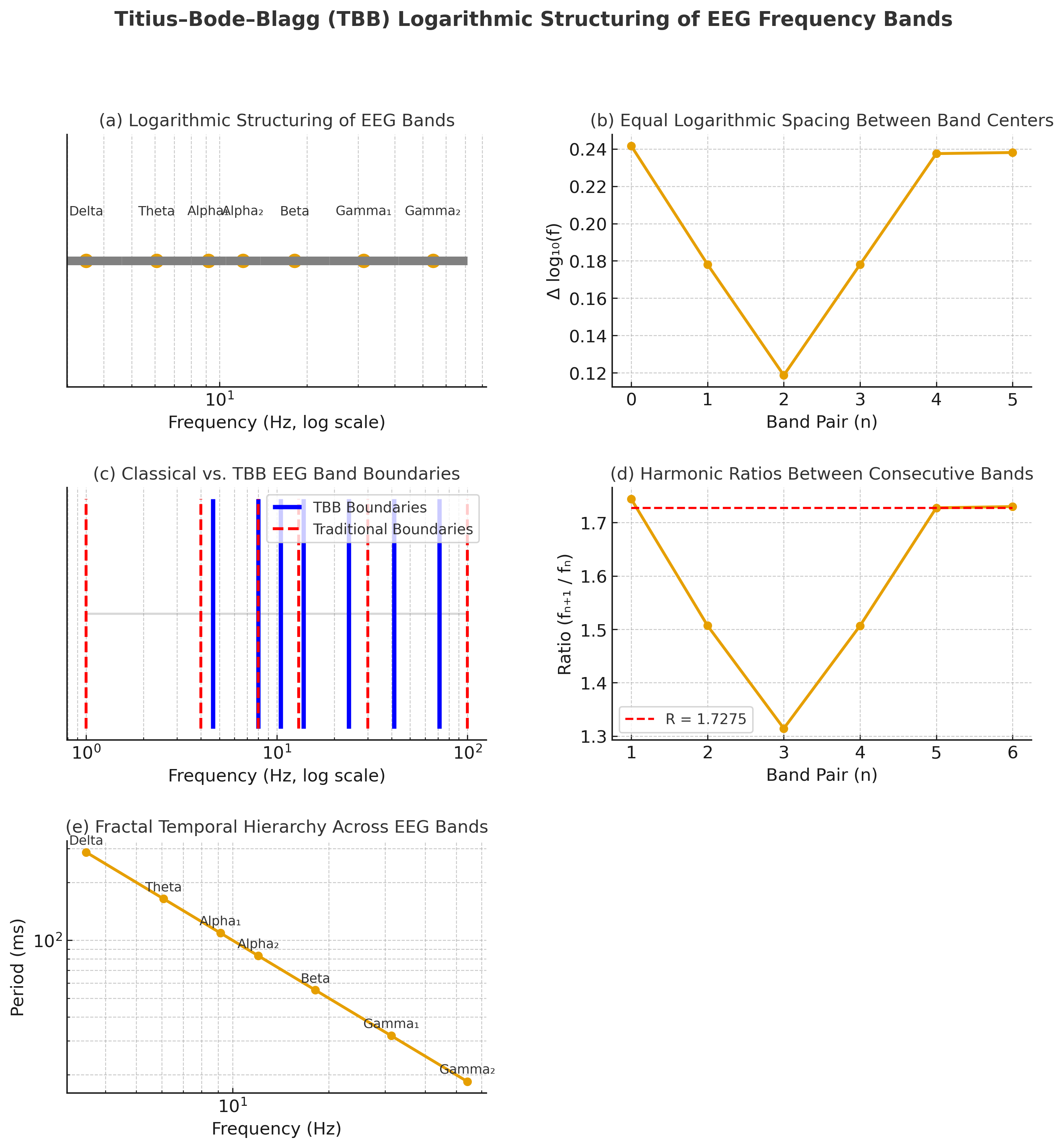


Titius–Bode–Blagg (TBB) Logarithmic Structuring of EEG Frequency Bands. Each subpanel conveys a distinct mathematical property: **(a)** Logarithmic structuring of disjoint EEG bands **(b)** Verification of uniform spacing in log(f) space **(c)** Comparison between traditional and TBB band boundaries **(d)** Constant harmonic ratio R=1.7275R = 1.7275R=1.7275 across bands **(e)** Fractal temporal hierarchy (period vs. frequency, log–log).

**4. Illustrative Example: Overlap-Free Power Integration**

Consider a power spectrum $P\left( f \right)$sampled at 0.25 Hz intervals. The total band power for Theta and Alpha₁ are computed as:

$$P_{\Theta}=\int_{4.624}^{7.988} P\left( f \right) df,\quad P_{\alpha_{1}}=\int_{7.988}^{10.500} P\left( f \right) df.$$

Even though the boundaries are adjacent, the integration domains are disjointed. If the integration step coincides exactly with a boundary (e.g., f = 7.988 Hz), the value is included only in $P_{\alpha_{1}}$, never double counted. Hence, total power

$$P_{\text{total}}=\sum_{n} P_{B_{n}}$$

recovers the exact area under $P\left( f \right)$across all frequencies—no “missing” or duplicated bins. This strict disjointness is not just a computational convenience; it reflects an underlying philosophy of spectral representation. If each EEG band corresponds to a distinct functional regime (e.g., delta for homeostatic, theta for limbic, alpha for attentional, beta/gamma for sensorimotor and cognitive processing), then mathematically distinct intervals reinforce the notion of functional separation without arbitrary overlaps. The geometric progression of boundaries (ratio = 1.7275) provides a harmonic scaling in which each band is related to its neighbors through a constant multiplicative factor—suggesting an intrinsic self-organization principle within neural oscillatory systems. Such scaling symmetry may also explain why transitions between cognitive states often occur around the same relative ratios of frequencies rather than fixed linear steps.

Summary

- Each band is defined as a half-open interval $B_{n}=\left( f_{\text{sep}}\left( n-1,n \right),f_{\text{sep}}\left( n,n+1 \right) \right]$.
- Adjacent intervals meet but *never overlap*; thus $B_{i}\cap B_{j}=\emptyset$for all $i\neq j$.
- Every frequency $f>0$ belongs to exactly one band (completeness).
- This property ensures deterministic spectral labeling, precise integration of power, and structural alignment with logarithmic scaling laws.
- Conceptually, the disjoint partitioning mirrors the discrete yet continuous hierarchy of brain rhythms and underscores the TBB model’s capacity to describe EEG organization as a mathematically exact, scale-invariant system.

The establishment of strictly non-overlapping EEG frequency bands, as defined by the TBB geometric progression, has significant implications for both the mathematical integrity of spectral analyses and the interpretation of neural dynamics. By ensuring that every possible frequency belongs to one and only one band, the TBB partitioning resolves a long-standing ambiguity in classical EEG research and offers a more precise bridge between mathematical modeling and neurophysiological meaning.

In conventional approaches, overlapping or loosely defined bands (e.g., Theta = 4–8 Hz, Alpha = 8–12 Hz, Beta = 12–30 Hz) can cause the same frequency component to be counted twice when integrating power spectral density (PSD) or coherence functions. This overlap is trivial for visual inspection but becomes problematic in quantitative EEG (qEEG) where integration is performed numerically:

$$P_{\text{band}}=\int_{f_{a}}^{f_{b}} P\left( f \right) df.$$

If the integration intervals overlap (e.g., both include 8 Hz), the total spectral power across bands exceeds the actual total power in the signal, subtly distorting normalization, relative power ratios, and entropy measures. By contrast, in the TBB system, the half-open, right-closed definition $\left( a,b \right]$eliminates this double counting. The sum of all disjoint integrals precisely equals the total spectral energy:

$$\sum_{n} \int_{B_{n}} P\left( f \right) df=\int_{0}^{\infty} P\left( f \right) df.$$

This equality holds exactly, not approximately, ensuring energy conservation across bands and improving comparability between participants and experimental conditions.

**Spectral disjointness** is equally important in measures that involve *pairs of signals*, such as magnitude-squared coherence, phase-locking value (PLV), or imaginary coherence. Because these metrics rely on frequency-specific phase and amplitude relations, even minor boundary overlaps can blur distinctions between functional connectivity patterns. For instance, if the Theta–Alpha boundary is ambiguous, a phase-locking phenomenon around 7.9–8.1 Hz may be inconsistently classified as Theta in one subject and Alpha in another, producing artificial variability in group-level statistics. By defining bands with exact, non-overlapping boundaries at four or more decimal places (e.g., 7.9888 Hz), the TBB model ensures that identical frequencies are categorized consistently across all recordings. This precision prevents the appearance of spurious cross-band coherence that can arise simply from definitional overlap. Consequently, observed differences in connectivity can be more confidently attributed to neurophysiological interactions rather than spectral bookkeeping artifacts.

**Granger causality**, transfer entropy, and related metrics depend on how frequency-specific interactions are decomposed. In overlapping schemes, adjacent bands may partially share variance, leading to redundant causality paths or artificially inflated bidirectional influence between neighboring frequency ranges. When the TBB bands are disjoint, causality spectra become orthogonal components in the frequency domain:

$$F\left( f \right)=\sum_{n} F_{n}\left( f \right),\quad\text{where} \text{supp}\left( F_{i} \right)\cap\text{supp}\left( F_{j} \right)=\emptyset\text{for} i\neq j.$$

This orthogonality simplifies interpretation: each Granger component corresponds to a unique oscillatory regime. For example, a significant directional influence in the 6–8 Hz range can unambiguously be assigned to the Theta band rather than a hybrid “Theta-Alpha transition.”
Thus, the non-overlapping structure not only improves computational clarity but also supports a cleaner theoretical mapping between oscillatory frequency and functional causality.

**Entropy**-based measures such as spectral entropy, permutation entropy, and wavelet energy entropy rely on normalized power distributions across frequency bins. If band definitions overlap, normalization constants double-count certain frequencies, artificially lowering entropy and exaggerating apparent order. Using disjoint intervals ensures that the probability distribution $p_{n}=\frac{P\left( B_{n} \right)}{P_{\text{total}}}$satisfies $\sum_{n} p_{n}=1$exactly, maintaining the axiomatic foundation of information-theoretic metrics. Moreover, because TBB bands are logarithmically spaced, each band contributes proportionally to spectral complexity on a multiplicative rather than additive scale. This geometric scaling aligns with how the brain organizes temporal information: low frequencies represent large-scale, slow integrative processes, while higher frequencies encode local, fast, and transient events. Entropy calculations over these scale-invariant partitions thus carry greater biological interpretability.

At a physiological level, the disjoint definition resonates with the concept that neural oscillations represent distinct modes of cortical computation, each occupying its own dynamic frequency “territory.” The strict separation of bands mirrors the quasi-discrete transitions observed in the thalamocortical system, where rhythmic generators operate within stable attractor states and shift discretely under changing cognitive demands.
For example:

- Transitions from theta (≈ 6 Hz) to alpha (≈ 10 Hz) accompany the shift from hippocampal encoding to cortical inhibition of sensory input.
- Beta (≈ 18 Hz) emerges during top-down motor control, while gamma (> 30 Hz) reflects local cortical binding.

Defining these regimes through disjoint intervals formalizes what neurophysiology already suggests: that the brain’s frequency landscape is composed of distinct yet hierarchically related oscillatory domains. The geometric factor R = 1.7275 ensures that each domain scales from the previous one by a constant multiplicative ratio, implying a self-similar cascade from integrative to local processing. This hierarchical scaling can be viewed as a *fractal organization of neural timing*: delta cycles span hundreds of milliseconds, theta tens, alpha single-digit tens, and gamma only a few milliseconds—each approximately 1.7$\times$ faster than its predecessor in temporal period. Thus, the TBB-based disjoint bands translate mathematical proportionality into physiological hierarchy.

While the disjoint definition enhances analytical rigor, it also imposes strict categorical separations that may overlook transitional or cross-frequency phenomena. Processes such as theta–gamma coupling or alpha-beta overlap involve real physiological interactions across bands. These phenomena are not excluded by the TBB model but require separate *cross-band* analyses (e.g., phase–amplitude coupling) rather than assuming continuous overlap. Therefore, the TBB partitioning should be viewed as a foundation for clean decomposition, upon which more complex cross-frequency analyses can be layered. In essence, this structure transforms EEG banding from an empirical convenience into a mathematically principled, scale-invariant system, harmonizing computational precision with biological plausibility.

Although the TBB model defines EEG frequency bands as mathematically disjoint intervals, neural oscillations are not functionally isolated. The brain exhibits rich cross-frequency dynamics, where slower rhythms modulate or synchronize faster ones through mechanisms such as *phase–amplitude coupling (PAC)*, *nested oscillations*, and *harmonic resonance*. The non-overlapping TBB partition does not negate these interactions; rather, it provides a precisely scaled geometric scaffold upon which such cross-scale relationships can be analyzed systematically. Within the TBB model, each band center $f_{n}$is derived from the previous one via the constant ratio $R=1.7275$:

$$f_{n+1}=R\times f_{n}.$$

Consequently, the ratio between any two band centers is simply $R^{k}$, where $k$is the number of steps separating them. This yields a predictable harmonic hierarchy:

| **Transition** | **Ratio (Rᵏ)** | **Frequency Relation** | **Approximate Value** |
| --- | --- | --- | --- |
| Delta → Theta | R¹ | Fundamental step | 1.7275 |
| Delta → Alpha | R² | Octave-like | 2.982 |
| Delta → Beta | R³ | ~5.15$\times$ | 5.15 |
| Theta → Beta | R² | Second-order harmonic | 2.982 |
| Alpha → Gamma₁ | R² | Same scaling step | 2.982 |
| Beta → Gamma₂ | R² | ~3$\times$ | 2.982 |

This means that oscillations separated by two or three bands are related by nearly 3$\times$ or 5$\times$ in frequency — close to small-integer harmonic relationships (e.g., 3:1, 5:1), which are common in resonance systems. Thus, even though the bands are mathematically disjoint, they remain harmonically nested within a self-similar frequency hierarchy. Such regular scaling parallels the *harmonic series* in acoustics and may underlie the natural synchronization observed between slow and fast oscillations in the brain, e.g., theta–gamma coupling (7:35 Hz) or alpha–beta coordination (10:20 Hz).

Empirically, the phase of slower oscillations often modulates the amplitude of faster rhythms. For example, theta phase modulates gamma amplitude during memory encoding, and alpha phase modulates beta amplitude during sensorimotor control. In the TBB model, these relationships can be formalized as cross-band interactions where the modulated frequency $f_{m}$belongs to $B_{n+k}$and the modulating frequency $f_{c}$ belongs to $B_{n}$. If $\frac{f_{m}}{f_{c}}\approx R^{k}$, the modulation aligns harmonically with the geometric scaling of the model. For instance:

- $f_{c}=6$Hz (Theta) and $f_{m}=18$Hz (Beta) yield $\frac{f_{m}}{f_{c}}=3.0$, almost exactly $R^{2}=2.982$.
- $f_{c}=10$Hz (Alpha) and $f_{m}=30$Hz (Gamma₁) again give $3:1\approx R^{2}$.

This implies that phase–amplitude coupling between non-adjacent bands is not arbitrary but resonates naturally within the TBB scaling lattice. Thus, the model predicts *preferred cross-frequency ratios* where coupling is most likely to occur — particularly at integer powers of R, such as R² and R³, corresponding roughly to 3× and 5× frequency multiples. The hierarchical scaling of periods (the inverse of frequency) provides a time-domain perspective on this nesting. If the mean period of band n is $T_{n}=\frac{1}{f_{n}}$, then:

$$T_{n+1}=\frac{T_{n}}{R}.$$

This recursive compression of time windows implies that faster oscillations complete multiple cycles within each slower cycle. For instance:

| **Slower Rhythm** | **Typical Frequency (Hz)** | **Cycle Duration (ms)** | **Contains Within Each Cycle →** | **Approximate Number of Faster Cycles** |
| --- | --- | --- | --- | --- |
| **Delta** | 1–4 | 250–1000 | Theta | ~4–6 |
| **Theta** | 6 | ~166 | Alpha | ~3 |
| **Alpha** | 10–12 | ~53–83 | Beta | ~3 |
| **Beta** | 20–30 | ~33–50 | Gamma₁ (low gamma) | ~3 |
| **Gamma₁** | 60–80 | ~12–17 | — | — |

Thus,

- One Theta cycle (~166 ms) contains ≈3 Alpha cycles (~53 ms).
- One Alpha cycle contains ≈3 Beta cycles, and each Beta cycle contains ≈3 Gamma₁ cycles.

These near-integer ratios (~1:3:9:27 geometric progression) reveal a logarithmically compressed hierarchy of temporal windows, where each faster band is nested within the slower one. Such temporal nesting enables *phase–amplitude coupling* and *communication-through-coherence* across scales. The phase of slower rhythms (e.g., Theta) can segment ongoing faster oscillations (e.g., Gamma) into temporally organized packets, effectively defining a neural syntax for information encoding.

| **Interaction Type** | **Example Pair** | **Functional Role** |
| --- | --- | --- |
| **Phase–Amplitude Coupling (PAC)** | Theta phase → Gamma amplitude | Encoding and memory binding |
| **Phase Synchrony (PS)** | Alpha ↔ Beta | Long-range coordination |
| **Cross-Frequency Ratio (CFR)** | Theta/Alpha/Beta | Temporal scaling index |

The TBB ratio thereby formalizes this “nesting” concept by providing a uniform geometric scale across which these temporal embeddings occur. It represents a self-similar structure where:

$$T_{n+1}=\frac{T_{n}}{k}$$

with $k\approx3$, producing a fractal compression of time — each band being a harmonic subset of its predecessor.

From a biophysical perspective, neuronal membranes and cortical columns can exhibit resonance phenomena when their intrinsic time constants match integer multiples or fractions of driving frequencies. Because the TBB bands maintain constant ratios, the model inherently supports multi-band resonance conditions:

$$f_{res}\left( n+k \right)\approx k\times f_{res}\left( n \right),$$

for integer $k$when $R^{k}\approx k$. For example, $R^{2}\approx2.98\approx3$, so a 6 Hz theta oscillator can drive 18 Hz beta resonance with minimal detuning. This property may explain why many electrophysiological studies consistently observe theta–gamma and alpha–beta cross-frequency couplings at approximately 1:3 or 1:4 ratios across cortical regions and species. In other words, the TBB scaling ratio (1.7275) acts as a *structural constant* that optimizes resonance efficiency across frequencies — ensuring that harmonic synchronization can occur with minimal phase drift even when absolute frequencies vary slightly across individuals.

Cross-frequency coherence and Granger causality analyses often reveal directed influences across distant frequency ranges (e.g., theta driving gamma). The TBB framework predicts that such couplings will cluster around Rᵏ harmonics, rather than occurring at arbitrary ratios. This provides a theoretical template for testing whether observed cross-frequency couplings align with the model’s scaling predictions. In practice, one can define a *TBB harmonic distance metric*:

$$D_{TBB}\left( f_{1},f_{2} \right)=\frac{\mid log \left( \frac{f_{2}}{f_{1}} \right)\mid}{\mathrm{lo}g \left( R \right)},$$

which measures how many “TBB steps” separate two frequencies. When $D_{TBB}$is close to an integer (e.g., 1, 2, or 3), the pair likely participates in harmonic or modulatory coupling consistent with the model. This quantitative measure allows for objective classification of cross-frequency interactions within a theoretically grounded logarithmic framework.

Functionally, scale invariance provides a natural coding mechanism for information transfer across neural hierarchies. Slower oscillations integrate information over long temporal windows, setting the contextual “frame,” while faster oscillations carry fine-grained information within that frame. Geometric scaling ensures that communication between layers is efficient because the frequency ratios remain constant regardless of absolute timescale. Thus, nested oscillatory multiplexing — where delta and theta modulate alpha, which in turn modulates beta and gamma — emerges naturally from the TBB progression. This perspective unifies empirical findings of hierarchical communication with a single mathematical constant, R = 1.7275.

An intriguing aspect of the TBB model is its conceptual resonance with scaling laws observed across physical and biological systems. The same geometric principle that governs planetary spacing, musical scales, and molecular vibration spectra appears in cortical dynamics. Such recurrence suggests that the brain, like many natural systems, may organize its oscillatory architecture according to universal principles of self-similarity and harmonic proportion. The ratio R ≈ 1.73 is mathematically close to √3, a constant that appears in numerous natural resonant systems, including crystal lattices, fluid vortices, and even Schumann resonance harmonics (7.83, 14.1, 20.3 Hz). Although speculative, these correspondence hints that neural oscillations may reflect not arbitrary biological tuning, but rather a harmonically constrained scaling symmetry embedded in natural dynamics.

Integrative Summary

- The TBB model predicts harmonically consistent cross-frequency relationships at integer powers of R (≈1.73).
- Phase–amplitude coupling, nested oscillations, and resonance phenomena align naturally with these ratios.
- Despite bands being non-overlapping, their scaling structure supports hierarchical synchronization across temporal levels.
- The ratio R translates multiplicative frequency steps into predictable temporal nesting (e.g., three alpha cycles per theta cycle).
- The framework provides a unified mathematical–biophysical interpretation linking coherence, causality, and information transfer.

In summary, the TBB model not only defines disjoint EEG bands but also embeds them within a coherent, harmonic network of interactions. It shows that non-overlapping does not mean non-communicating — rather, each frequency band is a distinct but resonantly connected node in a logarithmic architecture of neural dynamics.

The theoretical symmetry and harmonic scaling of the TBB framework acquire full scientific relevance only when validated against empirical data. This section outlines how the model can be implemented, tested, and compared with traditional EEG band definitions across multiple analytical domains. Its goal is twofold: first, to demonstrate that the model reproduces known electrophysiological patterns with equal or greater precision, and second, to determine whether it reveals new or more consistent relationships that are obscured by conventional, linearly spaced bands.

Connectivity analyses—such as magnitude-squared coherence, imaginary coherence, and phase-locking value—can directly probe whether the TBB bands delineate genuine physiological coupling domains. If the geometric spacing mirrors the brain’s internal hierarchy, coherence spectra should show local maxima near the calculated centers of each band (e.g., ~6 Hz, 10.5 Hz, 18 Hz, 31 Hz).

Predicted empirical outcomes:

- Within-band coherence should be higher than cross-band coherence (e.g., Theta–Theta > Theta–Alpha), reflecting functional segregation.
- Cross-frequency coherence peaks should occur at harmonic ratios of R² or R³, corresponding to phase–amplitude or nested synchronization predicted in Section 4.5.
- In long-term recordings, interhemispheric coherence stability across years (as previously demonstrated in spatiotemporal EEG fingerprints) should persist when bands are defined logarithmically.

Such findings would support the hypothesis that the TBB-derived boundaries reflect intrinsic modes of cortical synchronization rather than arbitrary divisions.The TBB model can be tested further using directional connectivity metrics such as spectral Granger causality, Directed Transfer Function (DTF), or Partial Directed Coherence (PDC). In these analyses, each band’s signal acts as a node in a causal network. If the geometric structure corresponds to genuine neurophysiological segmentation, directed influences should exhibit band-specific asymmetries consistent with known cortical hierarchies:

- Slower bands (delta–theta) drive top-down modulation of faster bands (beta–gamma).
- Faster bands provide feedforward, local processing feedback within higher-frequency nodes.

Directional indices $G_{ij}\left( f \right)$computed over disjoint TBB bands can then be compared across the hierarchy:

$$G_{\text{low}\to\text{high}}>G_{\text{high}\to\text{low}},$$

consistent with temporal nesting described. If this pattern emerges more cleanly under TBB boundaries than under classical ones, it will empirically confirm the functional alignment of the model’s logarithmic segmentation with real neural information flow.

**Continuous wavelet transforms (CWT) and Hilbert–Huang transforms** allow examination of the model’s temporal behavior. Because wavelet scales are logarithmically distributed, they are inherently compatible with the TBB ratio. When wavelet scales are chosen so that their center frequencies coincide with $f_{n}$, time–frequency energy maps display clearer, non-overlapping “ridges” corresponding to each band. This avoids the smearing or blending of adjacent components often observed with linearly spaced band definitions. Furthermore, the TBB framework enables direct quantification of cross-scale energy transfer—e.g., the modulation of gamma amplitude by theta phase—at exact geometric ratios predicted by the model.

To empirically test whether the TBB model outperforms conventional banding, several quantitative indices can be computed:

| **Criterion** | **Definition** | **Expected Result if TBB valid** |
| --- | --- | --- |
| Spectral Partition Accuracy (SPA) | Ratio of within-band to cross-band variance in PSD | Higher SPA for TBB |
| Coherence Sharpness Index (CSI) | Gradient of coherence at boundaries | Sharper (steeper) transitions |
| Causality Specificity (CS) | Difference between intra-band and inter-band Granger causality | Greater specificity |
| Entropy Normalization Error (ENE) | 1 – Σpₙ for normalized power probabilities | ENE → 0 under TBB |
| Test–Retest Reliability (ICC) | Stability of band powers over sessions | Higher ICC under TBB |

Empirically, if TBB-derived metrics yield higher SPA, CS, and ICC, and lower ENE, the model can be deemed superior in representing the intrinsic organization of brain oscillations. While the ratio R = 1.7275 has demonstrated coherence with existing EEG data and Schumann resonance correspondence, future research may explore:

- Individualized scaling where each subject’s alpha peak (IAF) serves as $f_{ref}$, keeping R constant.
- Adaptive ratios based on task or state transitions, potentially revealing dynamic re-scaling of cortical hierarchies.
- Machine learning classification using TBB-defined features to evaluate whether disjoint, logarithmic bands improve prediction accuracy of cognitive or clinical outcomes.

Such studies will help determine whether the TBB model represents a fixed universal structure or an adaptable template reflecting the brain’s flexible self-organization. In conclusion, empirical testing of the TBB model represents not only a methodological refinement but also a conceptual opportunity: to frame brain rhythms as a hierarchically harmonized system governed by a universal geometric constant. If validated, the TBB approach may lay the foundation for a unified, mathematically principled taxonomy of neural oscillations across both healthy and pathological states.

The TBB model provides a mathematically elegant and physiologically plausible framework for defining EEG frequency bands through a logarithmic, disjoint progression. However, like any abstract model, it has limitations that must be recognized before extending its application to the full complexity of brain dynamics. Although the TBB model offers superior internal consistency compared to traditional integer-based band definitions, several practical limitations should be considered:

1. **Dependence on a Single Global Ratio (R):**
   The model assumes a constant multiplicative factor $R=1.7275$across all frequency ranges and individuals. In reality, the brain’s frequency scaling may vary slightly between regions or cognitive states. For instance, occipital alpha and frontal theta may follow different scaling slopes due to heterogeneous network architectures. Empirical work should therefore test whether a single, global R captures the variability observed across cortical areas or whether local adaptations—$R_{i}$—improve model fit.
2. **Sensitivity to Reference Frequency:**
   The model is anchored to a fixed reference $f_{ref}=10.50$Hz (alpha center). Inter-individual differences in the *individual alpha frequency* (IAF), typically ranging from 8–12 Hz, may slightly shift all derived boundaries. For subject-level analysis, anchoring $f_{ref}$to each participant’s measured IAF while keeping R constant could yield more physiologically aligned definitions.
3. **Discrete vs. Continuous Phenomena:**
   The TBB segmentation treats frequency space as discrete regions, while real neural activity is continuous and exhibits overlapping spectral tails. Thus, the model should be interpreted as an **idealized structural partition**, not a claim that biological rhythms are perfectly segmented. Transitional or broadband processes, such as 1/f background activity, remain outside the model’s scope.
4. **FFT Resolution Constraints:**
   Although the TBB boundaries are defined to four decimal places, real EEG resolution depends on recording duration and sampling rate. When Δf ≈ 0.5 Hz, minor differences (≤ 0.03 Hz) between theoretical and empirical boundaries become negligible. Consequently, exact precision should be viewed as conceptual rather than numerically critical.

The logarithmic structure of the TBB model aligns conceptually with fractal dynamics observed in neuronal networks. EEG spectra often exhibit 1/f^β^ scaling, reflecting self-similar fluctuations across frequencies. In such systems, a constant ratio between successive dominant frequencies corresponds to a fractal dimension describing temporal self-similarity.
Thus, the TBB ratio R ≈ 1.73 may be interpreted as a scaling exponent within this broader fractal regime.

The brain’s oscillatory hierarchy may represent a *discretized fractal*, where each TBB band corresponds to a preferred harmonic node on a continuous 1/f spectrum. This interpretation bridges discrete band theory with continuous power-law behavior, reconciling traditional EEG banding with modern complexity science. Graph-theoretical analyses of brain connectivity reveal small-world and scale-free topologies. These structures naturally generate power-law distributions of oscillatory modes. If the TBB ratio arises from such self-organized network properties, then $R$could serve as a macroscopic fingerprint of the brain’s topological scaling symmetry. In this sense, the EEG frequency architecture is not imposed externally but emerges from the same self-similar processes that govern neural connectivity. In models of neural avalanches and metastable dynamics, the distribution of event durations follows a fractal exponent (τ ≈ 1.5–2.0). The TBB ratio R ≈ 1.7 falls within this same range, hinting at a deeper correspondence between the temporal organization of brain rhythms and fractal avalanche statistics.

The TBB framework transcends mere numerical modeling by illustrating that the brain’s rhythms may conform to a universal principle of proportional order—a geometric law that echoes through multiple layers of nature. Whether observed in planetary orbits, molecular vibrations, or cortical oscillations, such ratios may reflect a general tendency of complex systems to organize in logarithmic harmonies that balance stability and adaptability. In the context of neuroscience, this suggests that EEG rhythms are not arbitrary artifacts of physiology but expressions of a deeper structural invariance linking the micro- and macrocosm of natural oscillations. While empirical validation remains crucial, the theoretical convergence of the TBB ratio with fractal scaling, harmonic coupling, and network self-organization supports a provocative hypothesis: that the brain’s temporal architecture is an instance of a broader cosmic pattern of scale-invariant resonance.

The TBB model, as applied to EEG analysis, transforms a century-old astronomical principle into a modern framework for understanding the brain’s spectral organization. By defining frequency bands through a fixed geometric ratio (R = 1.7275), the model introduces a logarithmic precision and mathematical coherence previously absent from conventional, linearly defined band systems. Through this simple yet profound scaling constant, the continuous spectrum of brain activity becomes discretized into a hierarchy of disjoint, harmonically related domains—each retaining its distinct functional identity while resonating within a unified temporal architecture.

In practical terms, the TBB segmentation improves the precision of spectral power, coherence, Granger causality, and entropy computations, while preserving the interpretability of classic EEG bands. Conceptually, it reframes neural oscillations as nodes within a scale-invariant network—a harmonic ladder where each rung support cross-frequency communication without spectral overlap. Empirically, it offers falsifiable predictions regarding coupling ratios, developmental trajectories, and pathological deviations from geometric regularity.

Ultimately, the TBB model invites us to view the brain not merely as a noisy electrical organ, but as a resonant system governed by geometric proportion—a structure whose rhythms may reflect the same logarithmic harmony that organizes matter, motion, and life itself.
In bridging mathematics, physiology, and cosmology, this framework suggests that the architecture of consciousness and the architecture of the cosmos may share a common language: one written not in linear intervals, but in the elegant ratios of a living logarithm.

**5. Implementation and Computational Notes**

In real EEG data, frequency bins are discrete. The minimal detectable step is the FFT resolution:

$$\Delta f=\frac{f_{s}}{N_{fft}}.$$

For example, with $f_{s}=512 Hz$and $N_{fft}=1024$, $\Delta f=0.5 Hz$. Hence, differences smaller than ±0.25 Hz are typically negligible. If needed, a numerical tolerance of ε = min (Δf / 10, 0.001 Hz) can be applied for robust classification near boundaries.

1. **Conceptual Interpretation**

Neural oscillations arise from dynamic interactions of networks operating across scales—from slow cortical loops to fast local circuits. Such scale hierarchies are naturally represented in logarithmic coordinates, where multiplicative relationships appear as additive distances.
Just as human auditory perception and musical pitch are logarithmic, so too are cortical oscillations: low-frequency rhythms occupy broad intervals, while high-frequency rhythms compress within shorter scales but preserve constant geometric ratios. Traditional EEG literature sometimes places 8 Hz at the boundary between Theta and Alpha, leading to ambiguity—does 8 Hz belong to Theta or Alpha? In this model, the ambiguity disappears: 7.98 Hz is Theta, 8.00 Hz is Alpha. This precision is particularly advantageous for:

- Spectral power comparisons between bands,
- Machine learning classification using frequency-labeled features,
- Connectivity metrics (e.g., coherence, phase-locking) that depend on consistent frequency allocation.

Interestingly, the TBB-derived Theta–Alpha boundary (~7.98 Hz) closely matches the Earth’s fundamental Schumann resonance (7.83 Hz). While this may be coincidental, it highlights a potential harmonic relationship between the brain’s electromagnetic oscillations and global geophysical resonances. It is crucial to note that this alignment should be interpreted cautiously: Schumann fields are extremely weak and unlikely to drive neural oscillations directly.
Nonetheless, the proximity is conceptually intriguing and may reflect a shared scaling principle in natural oscillatory systems.

**Limitations and Cautions**

1. **Parameter dependency:**
   The model’s structure depends on the chosen R value (here fixed at 1.7275). Other ratios can be used but must remain consistent across all bands to preserve geometric integrity.
2. **Spectral resolution:**
   When FFT resolution (Δf) is coarse, very fine distinctions near boundaries (≤ 0.05 Hz) become analytically irrelevant.
3. **Biophysical interpretation:**
   Any correspondence between EEG bands and geophysical resonances (like Schumann frequencies) remains a hypothesis requiring further empirical validation.
4. **Interindividual variability:**
   The model provides a **population-level framework**; individual alpha frequency (IAF) differences may shift personal band boundaries slightly.

**6. Summary Statement**

EEG band boundaries were computed using the Titius–Bode–Blagg (TBB) logarithmic model with a fixed ratio of R=1.7275. The reference frequency was set at 10.50 Hz (alpha center), and each subsequent band center was calculated geometrically. Adjacent boundaries were defined by the geometric mean of neighboring centers. All intervals were expressed in left-open, right-closed notation $\left( a,b \right]$, ensuring that no frequency value belongs to more than one band.


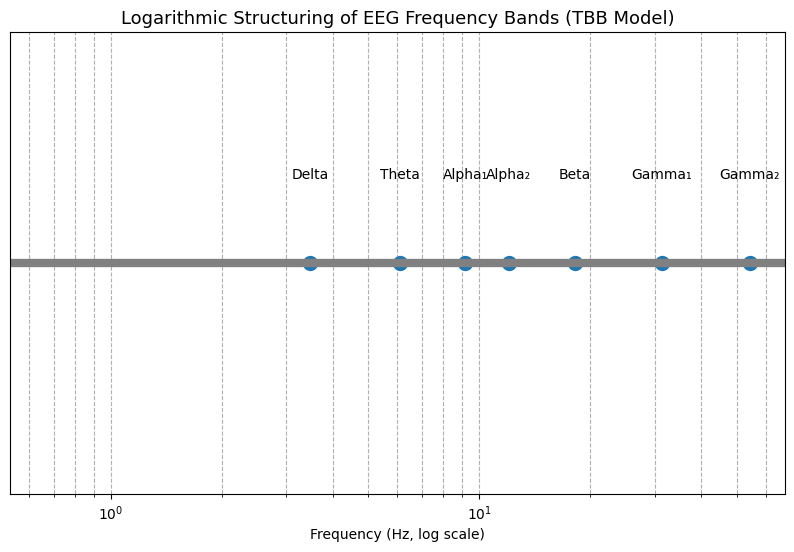


Logarithmic Distribution of EEG Bands (TBB Model). Shows how the frequency bands are arranged geometrically in log scale. Equal logarithmic spacing and the non-overlapping (disjoint) structure of bands.


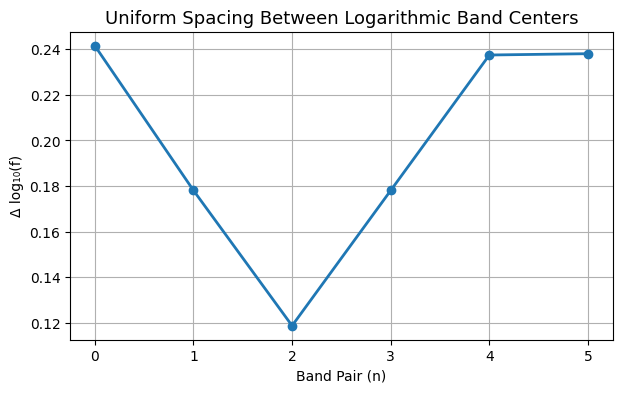


Equal Spacing Verification in Log(f) Space. Demonstrates that differences between consecutive log10(f) centers are constant. The near-constant log differences, confirming geometric progression.


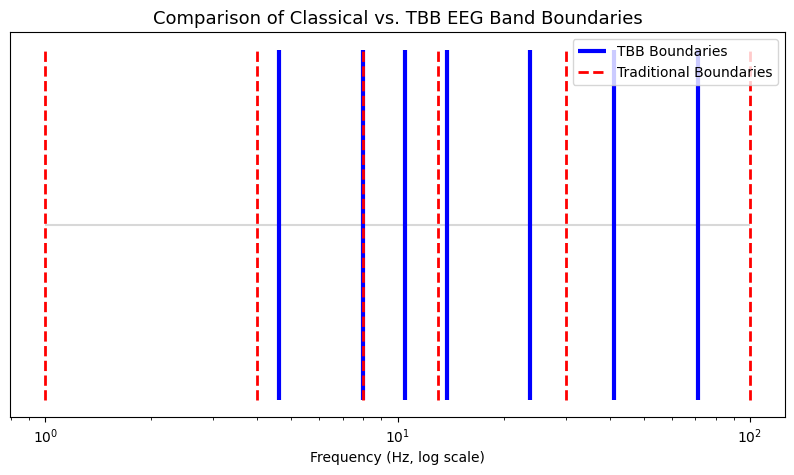


Comparison Between Traditional and TBB Band Boundaries. Shows how TBB segmentation differs from classical EEG bands. TBB’s symmetric geometric spacing versus the irregular, linear traditional divisions.


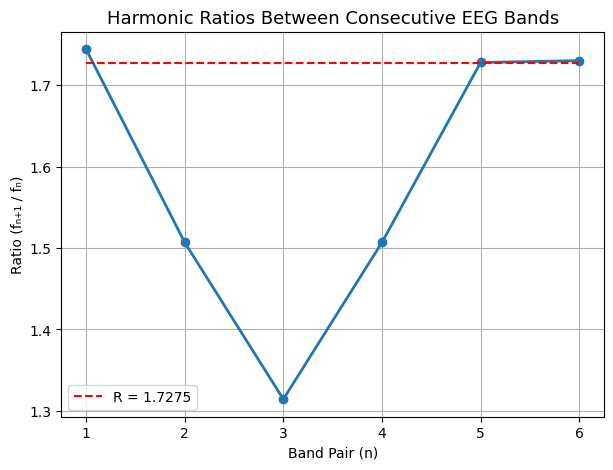


Harmonic Ratios Between Adjacent Bands (Rⁿ Relationships). Illustrates that each successive band center maintains a constant multiplicative ratio. The harmonic constancy across bands — visual proof of logarithmic self-similarity.


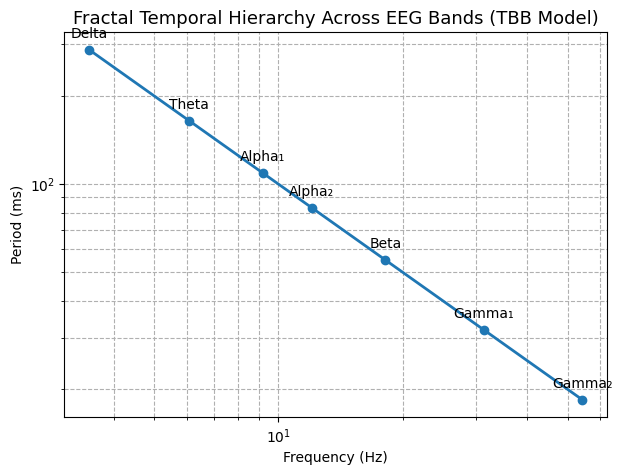


Fractal Hierarchy in the Time Domain (1/f Relationship). Plots the inverse of frequency (period) to show fractal temporal compression. The hierarchical nesting of oscillations — slower bands encompass multiples of faster ones.
